# Insights Into the Phylogenetic Distribution, Diversity, Structural Attributes, and Substrate Specificity of Putative Cyanobacterial Orthocaspases

**DOI:** 10.3389/fmicb.2021.682306

**Published:** 2021-07-02

**Authors:** Samujjal Bhattacharjee, Surbhi Kharwar, Arun Kumar Mishra

**Affiliations:** Laboratory of Microbial Genetics, Department of Botany, Institute of Science, Banaras Hindu University, Varanasi, India

**Keywords:** cyanobacteria, horizontal gene transfer, orthocaspases, phylogeny, programmed cell death

## Abstract

The functionality of caspase homologs in prokaryotic cell execution has been perceived, yet the dimensions of their metabolic pertinence are still cryptic. Here, a detailed *in silico* study on putative cyanobacterial caspase homologs, termed orthocaspases, in a sequenced genome of 132 strains was performed. We observed that 473 putative orthocaspases were distributed among 62% cyanobacterial strains subsumed within all the taxonomical orders. However, high diversity among these orthocaspases was also evident as the conventional histidine–cysteine (HC) dyad was present only in 72.03% of orthocaspases (wild-type), whereas the rest 28.18% were pseudo-variants having substituted the catalytic dyad. Besides, the presence of various accessory functional domains with Peptidase C14 probably suggested the multifunctionality of the orthocaspases. Moreover, the early origin and emergence of wild-type orthocaspases were conferred by their presence in *Gloeobacter*; however, the complex phylogeny displayed by these caspase-homologs perhaps suggested horizontal a gene transfer for their acquisition. However, morpho-physiological advancements and larger genome size favored the acquisition of orthocaspases. Moreover, the conserved caspase hemoglobinase fold not only in the wild-type but also in the pseudo-orthocaspases in *Nostoc* sp. PCC 7120 ascertained the least effect of catalytic motifs in the protein tertiary structure. Further, the 100-ns molecular dynamic simulation and molecular mechanics/generalized born surface area exhibited stable binding of arginylarginine dipeptide with wild-type orthocaspase of *Nostoc* sp. PCC 7120, displaying arginine-P1 specificity of wild-type orthocaspases. This study deciphered the distribution, diversity, domain architecture, structure, and basic substrate specificity of putative cyanobacterial orthocaspases, which may aid in functional investigations in the future.

## Introduction

Caspases are specialized cysteine-aspartate proteases, recognized to mediate apoptosis in metazoans. These peptidases are synthesized as inactive proenzymes, which upon activation attain dimeric configuration, having p20 and p10 sub-domains in each monomer (Earnshaw et al., 1999; Fuentes-Prior and Salvesen, 2004). The proteolytic activity of caspases is conferred by the p20-subunit that harbors a critical histidine–cysteine (HC) dyad in its active site, which cleaves target proteins after aspartate (D) residue, while the p10 subunit has a regulatory role. Although the caspases are generally harbored by the metazoans, its homologs are abundantly found in lower life forms. Pioneering studies revealed the presence of caspase homologs not only in single-celled eukaryotes (Uren et al., 2000) but also in bacteria (Aravind and Koonin, 2002), portraying the possibility of caspase-homolog-dependent PCD in these life forms.

Caspase homologs are a structurally diverse group of proteases, belonging to the Peptidase C14 family which also subsumed the archetype caspases. This family of proteases is characterized by the presence of a caspase-hemoglobinase fold (CHF) composed of four parallel β-sheets surrounded by three α-helices (Aravind and Koonin, 2002). Further, these homologs are broadly classified into paracaspases (PCAs) (Jaworski and Thome, 2016), metacaspases (MCAs) (Tsiatsiani et al., 2011; Minina et al., 2017), and OCAs (Klemenčič and Funk, 2018), based on their protein architecture. While PCAs and OCAs are devoid of p10-like subunit, the MCAs consist of both the sub-domain homologs. Although the catalytic HC dyad was conserved among caspase homologs, the aspartate P1 cleavage specificity annotated for all the Peptidase C14 members was absent in them. PCAs are arginine (R)-specific Ca^2+^-independent dimeric proteases (Hachmann et al., 2012), whereas MCAs are Ca^2+^-dependent monomers which specifically cleave after either R or lysine (K) residues (Hander et al., 2019). Additionally, the OCAs are speculated to have specificity for the basic residues but their detailed structural and functional aspects are yet to be unveiled (Minina et al., 2020). Owning to the significance of PCD in prokaryotes, a metagenomic study in the Baltic Sea exhibited the occurrence of Peptidase C14-containing proteins only among 4% of all the bacterial population (Asplund-Samuelsson et al., 2016). This 4% of bacteria mostly subsumed the members of actinomycetes, α-, β-, and γ-proteobacteria, and cyanobacteria (Koonin and Aravind, 2002; Asplund-Samuelsson et al., 2012). Most of the Peptidase C14-containing proteins in cyanobacteria lacked the p10-like sub-domain (Choi and Berges, 2013) and therefore considered as the OCAs, whereas only 4% accounted for putative MCAs (Klemenčič et al., 2019). An initial study on 33 whole-genome-sequenced cyanobacterial strains depicted the uneven distribution of OCAs, as complex strains with larger genome favored to acquire them (Jiang et al., 2010). Moreover, the substrate specificity of putative OCA, MaOC1, for RR dipeptide and Ca^2+^-independent autocatalysis was also demonstrated in *M. aeruginosa* PCC 7806 (Klemenčič et al., 2015). However, the identification of various pseudo-variants of caspase homologs lacking the functional HC dyad increased the obscure underlining of the dimension of their functionalities. In *Trypanosoma brucei*, the catalytic HC dyad was substituted by the YS (tyrosine–serine) motif in MCAs TbMC1 and TbMC4, probably rendering their proteolytic activity (Proto et al., 2011). The abundance of pseudo-OCAs in cyanobacteria having substituted catalytic motifs may suggest a wider applicability of these proteases (Klemenčič et al., 2019).

This study focuses on the functionality of wild-type OCAs (OCA having an HC catalytic dyad) and their physiological congruity in cyanobacteria. Here a comprehensive bioinformatics study has been conducted to describe the distribution, diversity, abundance, domain configuration, and phylogeny of 473 putative cyanobacterial OCAs. Additionally, the structural analysis of a wild-type OCA from *Nostoc* sp. PCC 7120 was performed using homology modeling and compared elaborately with their pseudo-counterparts. Further, docking and molecular dynamic (MD) simulation of the protein-substrate complex was performed to ensure the substrate specificity of this OCA. In addition, the binding free energy (ΔG_*bind*_) calculated using the molecular mechanics/generalized born surface area (MMGBSA) also assured the stability of the complex. This study will provide an updated and holistic view on the attributes of putative OCAs and may pave new ways to answer the existence of OCA-mediated PCD in cyanobacteria.

## Materials and Methods

### Retrieving the Sequences of Putative Cyanobacterial OCAs

The protein sequences of putative cyanobacterial OCAs were retrieved from the NCBI database^1^. The p20-like sub-domain (Peptidase C14)-containing protein sequences in 132 whole-genome-sequenced cyanobacterial strains were identified by performing domain enhanced lookup time accelerated (DELTA) BLAST (Boratyn et al., 2012), using 4AFR (MCA 2) of *Trypanosoma brucei* (Q585F3.1) as the query. Sequences with incomplete Peptidase C14, either at the N- or C-terminal, or lacking the domain, were manually eliminated. Finally, 473 putative OCA sequences from 82 cyanobacterial strains were further analyzed.

### Analyzing the Diversity, Distribution, and Abundance of Putative Cyanobacterial OCAs

All the protein sequences were manually analyzed and subsequently categorized into true (wild-type) and pseudo (mutated) OCAs, based on the catalytic dyad forming the active site. Only those proteins with classical HC dyad were considered as wild-type, whereas proteins having any other amino acid residues in the catalytic dyad were regarded as pseudo-OCAs. Further, the distribution and abundance of all OCA subtypes (true and pseudo OCAs) along the taxonomical order of these 82 cyanobacterial strains were compared against their genome sizes and morphological complexities.

### Domain Configuration of Putative Cyanobacterial OCAs

The full-length OCA sequences were subjected to CD search^2^ (Marchler-Bauer et al., 2017) against the Pfam database (threshold E value = 0.01) (Mistry et al., 2021). CD-search outputs were analyzed, and only specific hits for conserved functional domains were considered since they were top-ranked hits and conferred very high confidence, while superfamily hits were averted. Additionally, transmembrane domains (TM) were identified using TMHMM v2.0^3^ (Krogh et al., 2001). The number of different accessory domains that occurred in 473 putative OCAs was determined; however, successive repeats of domains within the same protein were disregarded. The domain architecture of few OCAs with frequently appearing accessory domains was visualized by Illustrator of Biological Sequence (IBS) v1.0 (Liu et al., 2015). Domain configurations for all the OCAs are enlisted in Supplementary Table 1.

### Multiple-Sequence Alignment and Phylogeny

Multiple-sequence alignment (MSA) and phylogeny were performed using only the Peptidase C14 domain of all the putative OCAs. Additional domains associated with the proteases were not considered to avoid any possible uncertainty due to their varied origin and evolutionary history. Four hundred seventy-three Peptidase C14 domains were aligned by MUSCLE (Edgar, 2004), in MEGA v7.0 using default parameters, i.e., gap open penalty = −2.9; gap extended penalty = 0; and hydrophobicity multiplier = 1.2 (Kumar et al., 2016). Subsequently, the features of these sequences were displayed as the Hidden Markov Model (HMM) logo using Skylign^4^ (Wheeler et al., 2014).

A rooted phylogenetic tree comprising 473 Peptidase C14 sequences was constructed by the neighbor-joining method (Saitou and Nei, 1987) using MEGA v7.0. The MCA-like protein from *Thalassiosira pseudonana* CCMP1335 (XP_002295354.1) was used as the outgroup, since it is a marine diatom and possesses a closer evolutionary relationship with cyanobacteria (Jiang et al., 2010). The branch lengths were in the same units as those of the evolutionary distances. The evolutionary distances were calculated using the Jones–Taylor–Thornton (JTT) matrix-based method (Jones et al., 1992). The reliability of each branch was tested with 1,000 bootstrap replications. All positions containing gaps and missing data in the MSA file were eliminated during tree construction. Distinct clades were expanded and viewed separately for better inference (Supplementary Figures 1–4). The bootstrap value (above 50) and sequence details for each node were also provided in Supplementary Figures 1–4.

### Homology Modeling and Structural Analysis

Phylogenetic analysis displayed YS and YN as the most prominent catalytic dyad substitutions, which otherwise constituted of HC in the wild-type OCAs. Therefore, the theoretical modeling was performed to delineate and compare the structures of wild-type (HC) and mutated (YS and YN) OCAs. Since *Nostoc* sp. PCC 7120 is a model cyanobacterium and not only harbored the wild-type (HC) OCAs but also consisted of other two abundant forms of pseudo OCAs, i.e., YS and YN types, therefore, three OCAs, i.e., WP_010999143.1 (HC), WP_010999263.1 (YS), and WP_010997820.1 (YN), of *Nostoc* sp. PCC 7120 were selected. Due to the lack of suitable templates, only the Peptidase C14 domains of WP_010999263.1 (YS) and WP_010997820.1 (YN) were modeled. High-quality homology models were constructed by Swiss-Model^5^ using default settings (Guex et al., 2009; Benkert et al., 2011; Bertoni et al., 2017; Bienert et al., 2017; Waterhouse et al., 2018; Studer et al., 2020). Further, template-based refinement and energy minimization of models were done using ModRefiner^6^, for which ideal templates for each model with high resolution, sequence identity, and maximum query coverage were retrieved from Swiss-Model (Supplementary Table 2; Xu and Zhang, 2011). The final models were validated by Ramachandran plot using PROCHECK^7^ (Laskowski et al., 1993) and ModFOLD8^8^ (Maghrabi and McGuffin, 2017; Supplementary Table 3). Additionally, the folds and topologies of these OCAs were generated by PDBsum^9^ (Laskowski, 2001). The properties of OCA models were analyzed and visualized by UCSF Chimera (Pettersen et al., 2004).

### Analyzing the Substrate-Binding Efficacy of Wild-Type OCA

Two oligopeptides, i.e., arginyl-arginine (RR) and aspartyl-glutamyl-valyl-aspartate (DEVD), were used to study the substrate specificity of wild-type OCA (HC; WP_010999143.1). While DEVD is a classical caspase substrate, RR was previously used as a substrate for cyanobacterial OCA, MaOC1 (Klemenčič et al., 2015). For the docking analysis, the substrate and the protease were treated as ligand and receptor, respectively. Docking was performed with Biovia Discovery Studio 2019 using the CDOCKER program (Wu et al., 2003). CDOCKER is a powerful grid-based molecular docking method that uses Chemistry at Harvard Macromolecular Mechanics (CHARMm). Here, the ligand is allowed to flex while the receptor is held rigid during the refinement. The docked complex was visualized using UCSF Chimera 1.13.1, and LigPlot+ was used to identify the substrate-interacting residues (Laskowski and Swindells, 2011).

### Simulation of the HC–RR Complex

The HC–RR complex was subjected to all-atom MD simulation using the Amber16 package (Case et al., 2016). The initial structure was prepared in the tleap module of Amber16, utilizing the ff14SB force field (Maier et al., 2015). The ligand force-field parameters were generated using antechamber (Wang et al., 2006) and gaff (Wang et al., 2004); also, the AM1-bcc method was used to generate charges (Jakalian et al., 2002). The simulation box was solvated by the TIP3P water model with a padding distance of 12 Å (Jorgensen et al., 1983), and NaCl was used as counter-ion in neutralizing the total charge of the system. Joung and Cheatham ion parameters were used for Na^+^ and Cl^–^ ions (Joung and Cheatham, 2008). The electrostatic interaction was calculated by the particle mesh Ewald (PME) method (Darden et al., 1993). Further, the SHAKE algorithm was used to restrain the binds involving hydrogen (Ryckaert et al., 1977) while the Langevin dynamics (Wu and Brooks, 2003) and Berendsen barostat (Berendsen et al., 1984) were utilized for temperature and pressure control, respectively.

To minimize the energy of the complex, initially, the HC–RR complex was restrained and only the solvent was minimized. Thereafter, the restraint potential was removed from the complex and the entire system was minimized. Following the minimization, gradual heating of the systems was performed at NVT with a 100-kcal restraint potential on the complex, and the temperature was increased to 300K in 300 ps. After heating, each system was subjected to equilibration for 1.8 ns in six cycles of 300 ps in which the restraint potential was subsequently reduced from 100 to 0 kcal/mol (100, 50, 20, 5, 0.5, 0 kcal/mol) at NPT. After equilibration, a production run of 100 ns was performed each at the NPT ensemble. The trajectory was visualized by VMD 1.9.3 and UCSF Chimera 1.13.1.

### Calculation of Binding Free Energy

The last 90-ns trajectory was used to calculate the binding free energy of the HC–RR complex (ΔG_*bind*_) using the Molecular Mechanics Generalized Born Surface Area (MMGBSA) method implemented in Amber16. The calculation was performed at the dielectric constant of 1 and 80 for solute and solvent, respectively (Ylilauri and Pentikäinen, 2013). The parameters developed by Onfriev et al. was used for GB-calculation (igb=5; α=1.0, β=0.8. γ=4.85) (Onufriev et al., 2004). Additionally, entropic contribution (ΔS) was not taken into account for determining ΔG_*bind*_. Other parameters were used as default.

## Results

### Distribution, Diversity, and Abundance of OCAs in Cyanobacteria

**FIGURE 1:**
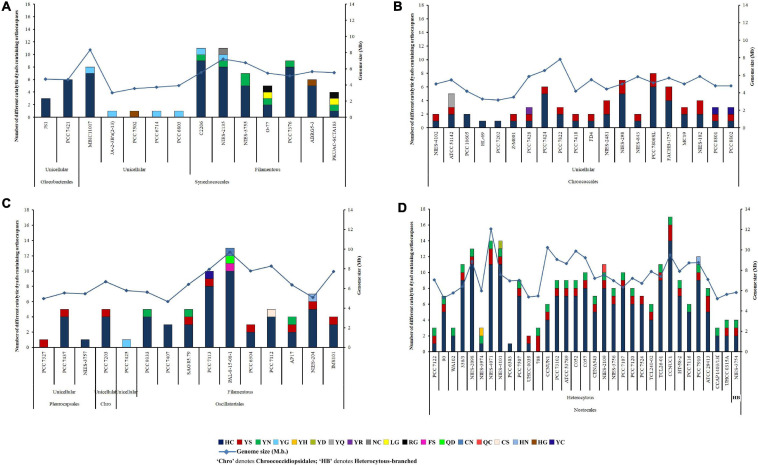
Abundance, distribution, diversity of putative cyanobacterial orthocaspases among **(A)** Gloeobacteriales, Synechococcales, **(B)** Chroococcales, **(C)** Pleurocapsales, Chroococcidiopsidales, Oscillatoriales, and **(D)** Nostocales. Cyanobacterial strains subsumed within different taxonomical orders are also categorized into unicellular, filamentous, and heterocytous branches based on their morphology. The full forms of the abbreviated strain names are mentioned in Supplementary Table 4.

An iterative analysis of 132 whole-genome-sequenced cyanobacteria resulted in the identification of 473 putative OCAs, harbored by 82 strains, accounting for about 62% of the query set (50 cyanobacteria either lacked OCAs or possessed incomplete sequences of Peptidase C14 domain). Out of 473 OCAs, 340 were wild-type (72.03%), having the HC dyad in their active sites, whereas the rest 133 (28.18%) were pseudo-variants. Among the pseudo-OCAs, YS- and YN-substituted catalytic motifs were predominant, accounting for approximately 47% and 31% of all pseudo-variants, respectively. Besides, 16 other pseudo-variants were also identified which comprised YG, YC, YQ, LG, RG, HN, HG, YH, YD, YR, NC, FS, QD, CN, QC, and CS motifs in their active sites (Supplementary Table 4). Among 82 cyanobacteria, few strains, i.e., *Gloeobacter kilaueensis* JS1, *Gloeobacter violaceus* PCC 7421, *Cyanobacterium aponinum* PCC 10605, *Cyanobacterium* sp. HL-69, *Cyanobacterium stanieri* PCC 7202, *Stanieria* sp. NIES-3757, *Geitlerinema* sp. PCC 7407, and *Calothrix* sp. PCC 6303, harbored only true OCAs. In contrast, *Synechococcus* sp. JA-2-3B’a(2-13), *Synechococcus* sp. PCC 7502, *Synechocystis* sp. PCC 6714, *Synechocystis* sp. PCC 6803, *Pleurocapsa* sp. PCC 7327, *Cyanothece* sp. PCC 7425, and *Nostoc azollae* 0708 harbored only pseudo-OCAs (Figure 1). However, at least one copy of both wild-type and pseudo-OCAs was identified in all other strains.

The abundance of OCA was estimated to be highest in Nostocales and Oscillatoriales with an average occurrence of 0.0921 and 0.0723 true OCA per 100 proteins, respectively. Besides, an abundance of 0.0416 true OCA per 100 proteins was observed in Chroococcales and the lowest abundance of 0.0145 true OCA per 100 proteins was estimated in Synechococcales. The average abundance of pseudo-OCAs was comparable for Nostocales (0.0349/100 proteins), Oscillatoriales (0.0264/100 proteins), and Chroococcales (0.0261/100 proteins), while the lowest abundance was recorded again for Synechococcales (0.0027/100 proteins) (Supplementary Table 4). The actual abundance of OCAs might obscure in Gloeobacterales, Pleurocapsales, and Chroococcidiopsidales due to the lesser number of whole-genome-sequenced cyanobacteria in them, yet OCAs harboring sequenced strains within these orders were examined.

Considerable variations in OCA frequencies were evident within the cyanobacterial orders. About 44% OCA-harboring strains (corresponding to 36 cyanobacteria), belonging to Gloeobacterales, Synechococcales, Chroococcales, Oscillatoriales, and Nostocales, displayed an exclusively high number of wild-type OCAs (≥5); however, the number of pseudo-OCAs ranged from 1 to 3 in 82 cyanobacteria. Nevertheless, the highest was observed in *Nostoc sphaeroides* CCNUC1 having 14 true and 3 pseudo OCA-encoding genes (Figure 1).

### Domain Variability and Configurations

Considerable variability in domain architecture among putative OCAs was observed due to the presence of multiple functional domains. Apart from Peptidase C14, 35 unique Pfam domains were identified in 473 OCAs (Table 1). While the true OCAs were predominantly multidomain proteins, most of the pseudo-OCAs lacked any identifiable domains, except Peptidase C14, showing a lesser domain variability than the wild type. Out of all the OCAs, a single Peptidase C14 domain was present in 34 (7%) and 123 (26%) true and pseudo OCAs, respectively, whereas 306 (65%) true and only 10 (2%) pseudo-OCAs harbored at least two functional domains (including Peptidase C14) (Supplementary Table 1). By disregarding the successive repeats of domains in a single protein, it was observed that the frequency of WD40 (26%) and AAA_16 (19%) was exceptionally high than that of other accessory domains. Moreover, FGE sulfatase (12%), GUN4 (9%), and Pentapeptide repeats (7%) also had a fairly high frequency. In contrast, 11 domains like TPR 19, Pentapeptide-4, AAA_22, GGDEF, CHAT, Ribosomal_L12, PBP-like_2, SAVED, TIR_2, PD40, and Pro-rich were associated with only one OCA each. Additionally, domains such as HEAT_2, TPR 11, HATPase_c, HisKA, DEAD, Helicase_C, TPR 16, TPR 12, TPR 2, Sel1, MAP7, PT, Clp_N, and EAL were also rare. Furthermore, few OCAs also consisted of domains of unknown function (DUF) (Table 1).

**TABLE 1:** Distribution of different accessory domains among putative true and pseudo-orthocaspases in cyanobacteria.

**S. no.**	**Accessory domains**	**True orthocaspase**	**Pseudo orthocaspase**	**Total**
				
1	WD40	82	1	83
2	AAA_16	60	–	60
3	FGE-sulfatase	38	1	39
4	GUN4	27	2	29
5	DUF4384	19	4	23
6	Pentapeptide	11	–	11
7	Peripla BP_6	8	–	8
8	TPR 1	4	1	5
9	HEAT_2	4	–	4
10	DUF2808	4	–	4
11	TPR 11	4	–	4
12	HATPase_c	4	–	4
13	HisKA	4	–	4
14	DEAD	3	–	3
15	Helicase_C	3	–	3
16	TPR 16	2	1	3
17	TPR 12	3	–	3
18	TPR 2	3	–	3
19	Sel1	2	–	2
20	DUF2610	2	–	2
21	MAP7	2	–	2
22	PT	2	–	2
23	Clp_N	2	–	2
24	EAL	2	–	2
25	TPR 19	1	–	1
26	Pentapeptide-4	1	–	1
27	AAA_22	1	–	1
28	GGDEF	1		1
29	CHAT	1		1
30	Ribosomal_L12	1		1
31	PBP-like_2	1		1
32	SAVED	1		1
33	TIR_2	1		1
34	PD40	1		1
35	Pro-rich	1		1
	Total	306	10	316

In the 473 putative OCAs, 67 consisted of transmembrane helices (TM), among which 30 and 37 were true and pseudo OCAs, respectively. The presence of one TM was fairly common among OCAs, but WP_015201768.1 (*Crinalium epipsammum* PCC 9333), WP_158517387.1 (*Moorea producens* PAL-8-15-08-1), and ABG51878.1 (*Trichodesmium erythraeum* IMS101) contained two TMs. Although the TM is generally located C-terminally to the Peptidase C14 domain, in AFZ48670.1 (*C. stanieri* PCC 7202), WP_015220159.1 (*C. aponinum* PCC 10605), AKV65607.1 (*Microcystis panniformis* FACHB-1757), WP_125731015.1 (*Microcystis viridis* NIES 102), WP_083305259.1 (*M. producens* PAL-8-15-08-1), and WP_167720235.1 (*Tolypothrix* sp. PCC 7910), TM was identified at the N-terminal with respect to Peptidase C14 (Supplementary Table 3).

**FIGURE 2:**
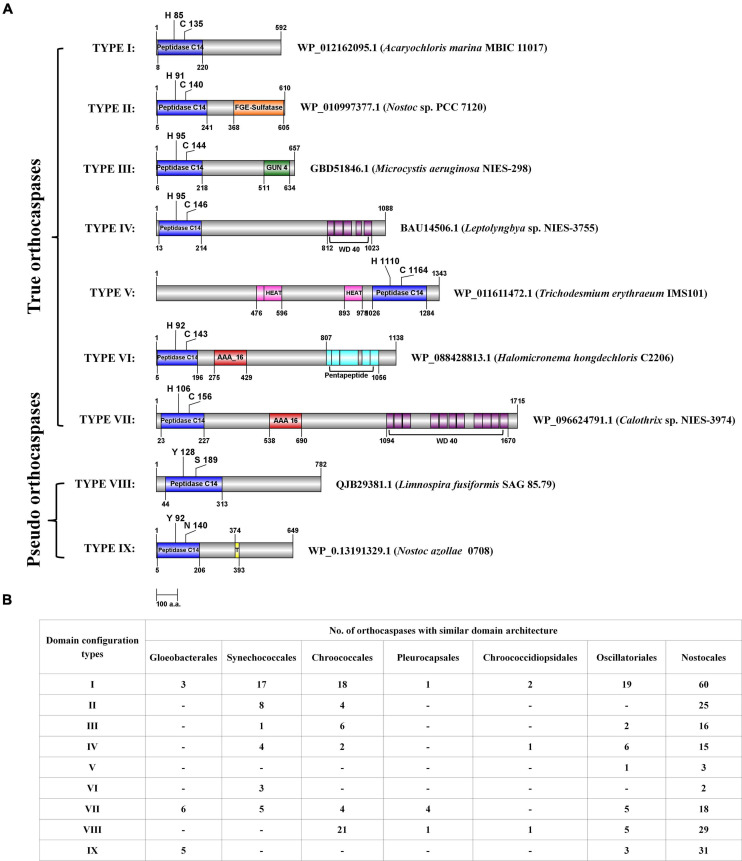
The domain configurations of few putative cyanobacterial orthocaspases with commonly observed accessory domains, and their distribution among taxonomical orders. **(A)** The domain configurations were categorized into nine different types, and a representative sequence was used to depict the position of domains for each type. Domain architectures obtained CD search using the Pfam database (*e*-value = 0.01). NCBI accession numbers and strain names are provided for the representative sequence. The figure is drawn to scale with numbers representing protein or domain length (amino acid numbers). Catalytic residues are indicated with a single-letter amino acid code with a position specifier. **(B)** The distribution of various domain configuration types among cyanobacterial orders is shown. Note that the configuration, i.e., the N- and C-terminal orders of domains, is the same in all the sequences sharing a particular type of domain architecture. However, the position of domain and catalytic residues shown in panel **(A)** may vary among the sequences.

Further, domain configuration revealed that the OCAs prevalently acquired a similar architecture. The common domain configurations attained by both wild-type and pseudo OCAs were classified into nine types (Figure 2A). In general, the Peptidase C14 domain was N-terminally located to the accessory domains in multidomain proteins (Figure 2); however, in Type V, the Peptidase C14 was located toward the C-terminal to HEAT_2 domain(s). Such architectures observed were WP_168163431.1 (*Calothrix* sp. 336/3), BAZ37947.1 (*Calothrix* sp. NIES 4101), WP_015119030.1 (*Rivularia* sp. PCC 7116), and WP_011611472.1 (*T. erythraeum* IMS101). Furthermore, it was observed that the OCAs of strains belonging to Nostocales consisted of all the domain configurations (Figure 2B).

### Multiple-Sequence Alignment and Phylogeny

**FIGURE 3:**
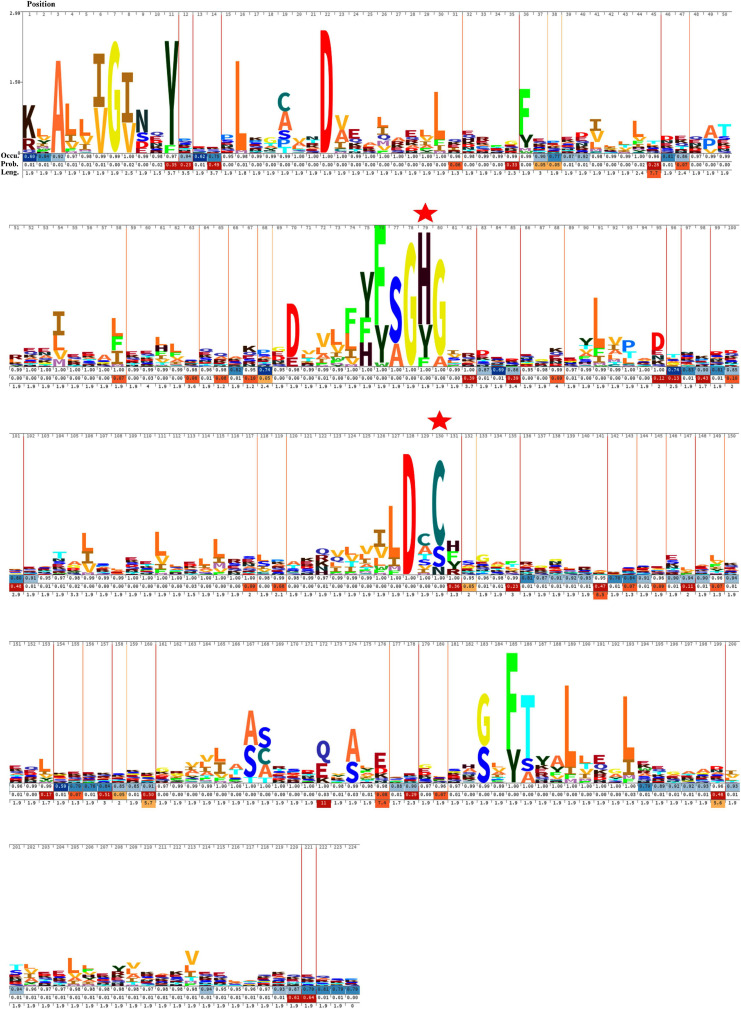
Hidden Markov Model (HMM) logos of the Peptidase C14 domain of putative cyanobacterial orthocaspases. Logos were built with 473 aligned Peptidase C14 domain sequences using Skylign. The values on the *Y*-axis correspond to the height of each single-letter amino acid code and reflected the probability of its occurrence at that specific position. The larger letter size indicates conserved residues among the protein sequences. The position of each amino acid residue is indicated at the top. The gap parameters, i.e., occupancy (Occu), insert probability (Prob.), and insert length (Leng.), are shown below each residue. The catalytic residues are indicated by “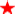.”

The sequence alignment of all OCA sequences displayed substantial diversity. The HMM logo exhibited a relatively conserved region surrounding the catalytic dyad (Figure 3). The consensus regions flanking the catalytic residues were (Y/F/H), (F/Y S/A), (G), (**H/Y/F),** (G/A), and (L/I/M/F), (D), (X), **(C/S/N)**, (H/F/Y/R) (catalytic residues are shown in bold). Besides, G7 and D27 were also conserved in all the OCA subtypes, probably suggesting their essentiality.

**FIGURE 4:**
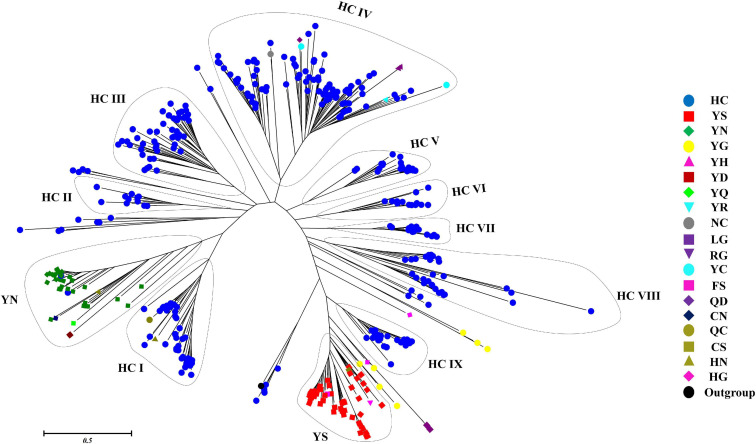
Phylogenetic relationship of putative cyanobacterial orthocaspases (OCAs). The rooted tree of only the Peptidase C14 domain of all the putative OCAs was constructed using the neighbor-joining method with the metacaspase-like protein of *Thalassiosira pseudonana* CCMP1335 (XP_002295354.1) as the outgroup. The optimal tree has the sum of branch length = 89.299. The tree is drawn to scale, with branch lengths in the same units as those of the evolutionary distances. The evolutionary distances were computed using the JTT matrix-based method and are in the units of the number of amino acid substitutions per site. The analysis involved 474 (473 cyanobacterial Peptidase C14 domain + 1 outgroup) sequences. All positions containing gaps and missing data were eliminated. Distinct clades are assigned based on the active site motifs in majority of the Peptidase C14 sequences. Eleven distinct clades were identified where HC catalytic dyads containing Peptidase C14 sequences formed nine whereas YS and YN catalytic motifs containing Peptidase C14 sequences formed two respective clades. Peptidase sequences with other substitution at catalytic motifs are scattered along the tree.

The phylogenetic relationship between 473 putative cyanobacterial OCAs was determined by an NJ tree constructed using their Peptidase C14 domains. Wild-type Peptidase C14 with HC dyad formed nine clades, whereas YS and YN motifs containing pseudo-variants were accumulated into two distinct clusters. Sequences with any other substituted active site motifs were scattered all along the phylogenetic tree (Figure 4). A thorough assessment of individual clusters revealed that most of the HC clades consisted of sequences from distantly related strains with variable lifestyles and morphologies (unicellular to heterocytous), except HC V and HC VII clades, harboring only multicellular strains of Nostocales and Oscillatoriales (Supplementary Figures 1–3). Evolutionary primitive strains, i.e., *G. kilaueensis* JS1 and *G. violaceus* PCC 7421 (Gloeobacteriales) having only wild-type Peptidase C14, were included in HC III and HC VIII clades. Moreover, pseudo-OCAs from Chroococcales, Pleurocapsales, Chroococcidiopsidales, Oscillatoriales, and Nostocales were harbored within the YS clade, whereas the YN clade subsumed only filamentous and heterocytous strains from Synechococcales, Oscillatoriales, and Nostocales (Supplementary Figure 4). Further, OCA-harboring strains of Nostocales showed the widest distribution, as they were present in all the clades.

### Structural Analysis of Cyanobacterial OCAs

**FIGURE 5:**
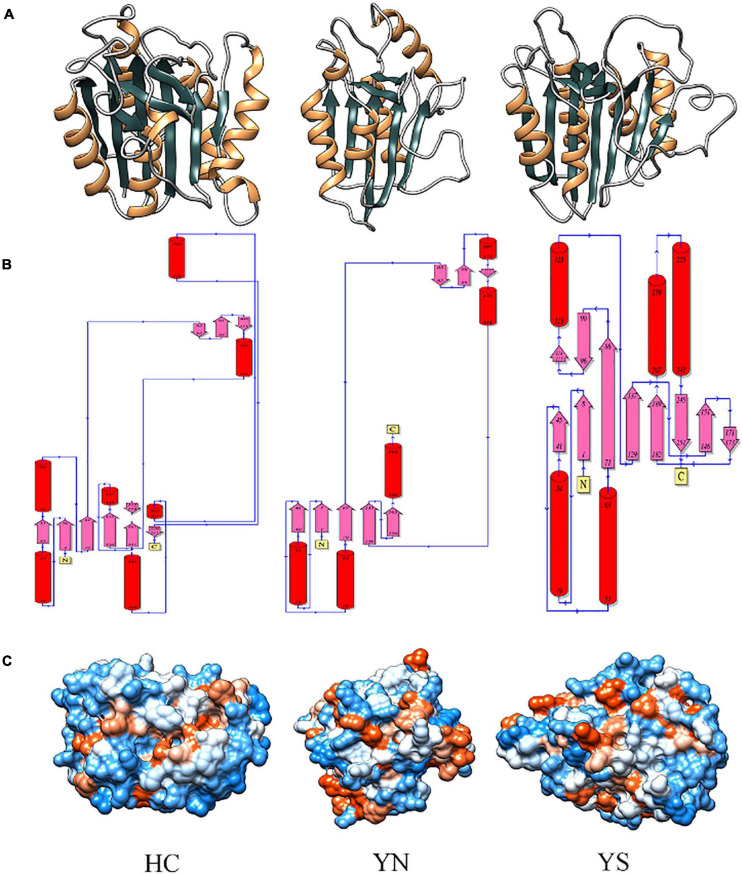
Comparing and analyzing the homology models of WP_010999143.1 (HC), WP_010997820.1 (YN), and WP_010999263.1 (YS) of *Nostoc* sp. PCC 7120, where HC is the wild-type OCA whereas YN and YS are pseudo orthocaspases. **(A)** 3D models of putative orthocaspases showing a conserved caspase-hemoglobinase fold (CHF). HC, YN, and YS possessed identical topology **(B)** due to conserved CHF with four parallel β-sheets and three α-helices. **(C)** Hydrophobicity surface view of modeled orthocaspases, where blue and orange colors represented hydrophilic and hydrophobic residues, respectively. Note that for WP_010997820.1 (YN) and WP_010999263.1 (YS), only the Peptidase C14 domain has been modeled due to template unavailability, while the full-length homology model was constructed for WP_010999143.1 (HC).

Homology modeling of WP_010999143.1 (HC), WP_010999263.1 (YS), and WP_010997820.1 (YN) from *Nostoc* sp. PCC 7120 displayed the presence of typical CHF (Figure 5A). Despite having different catalytic dyads, significant structural alterations among the three proteases were not evident. The core of these proteases comprised four parallel β-sheets, i.e., one N-terminal, two central, and one C-terminal sheets arranged in a 2–1–3–4 fashion, and three α-helices (one each after strands 1, 2, and between 3 and 4) (Figure 5B). However, only in the Peptidase C14 domain of WP_010999143.1 (HC) did the third and fourth β-sheets precede the proximal (H) and distal (C) active sites, respectively. Besides, more hydrophilic residues (blue) were present on the surface of all the three OCAs, conferring them as soluble proteins (Figure 5C).

### Analyzing the Substrate Specificity of HC

**FIGURE 6:**
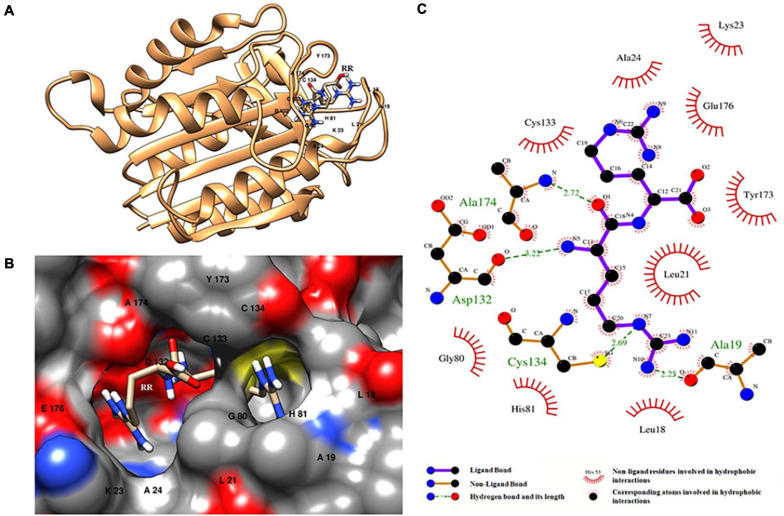
HC–RR complex formed upon docking RR dipeptide with wild-type orthocaspase, WP_010999143.1 (HC), of *Nostoc* sp. PCC 7120. **(A)** Binding position of substrate on the protein. **(B)** Binding pocket accommodating the substrate along with the interacting residues shown with a single-letter amino acid code and position specifier. **(C)** Interacting amino acid residues and the type of interaction with the substrate; amino acids are shown in a three-letter code with the position specifier.

**FIGURE 7:**
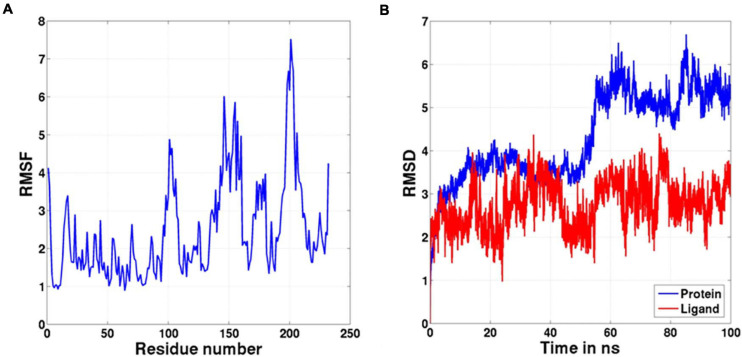
The graph representing **(A)** RMSF and **(B)** RMSD values of the complex during the 100-ns molecular dynamic simulation.

**FIGURE 8:**
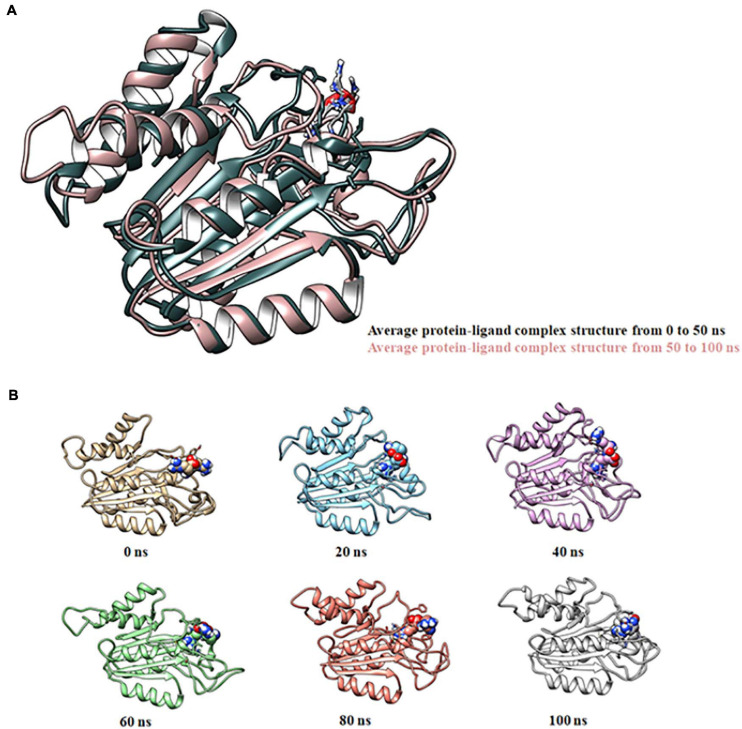
Molecular dynamic simulation of the HC–RR complex for 100 ns displaying its stability. **(A)** Superimposition of average complex structures between 0 and 50 ns and between 51 and 100 ns for determining the RMSD. RMSD was found to be 0.89 Å, suggesting minor deviation. **(B)** Snapshot of the HC-RR complex at 0, 20, 40, 60, 80, and 100 ns displaying stable incorporation of RR in the HC throughout the simulation time frame.

Molecular docking of HC with RR and DEVD revealed the binding of only RR with HC (Figure 6A), whereas no docked complex was formed between HC and DEVD (data not shown). The binding pocket for RR comprised L18, A19, L21, K23, A24, G80, D132, C133, Y173, A174, and E176 along with H81 and C134 (catalytic residues) (Figures 6B,C). Further, 100-ns simulation and MMGBSA illustrated the stability of the HC–RR complex. Residual fluctuation of the HC–RR complex depicted an average RMSF value of 2.45 ± 0.13 Å, among which residues between 18 and 97 and between 107 and 135 recorded lower fluctuations (Figure 7A). Most of the binding pocket residues were subsumed among these. Additionally, for 100-ns simulation the average RMSD of RR was 2.76 ± 0.54 Å, while the HC displayed two distinct trajectory: one from 0 to 50 ns with a lower average RMSD (3.43 ± 0.46 Å) and another from 51 to 100 ns with a higher average RMSD (5.21 ± 0.51 Å) (Figure 7B). However, upon superimposing the average structures, only an RMSD of 0.89 Å was observed, suggesting no major conformational changes in protein topology (Figure 8A). The presence of RR in the binding cavity of HC throughout the 100-ns simulation further conferred the specificity of HC for RR (Figure 8B). Additionally, ΔG_*bind*_ (= −16.99 ± 5.99) of the HC–RR complex also exhibited high binding efficacy (Table 2).

**TABLE 2:** Molecular mechanical energies combined with the generalized born and surface area continuum salvation (MMGBSA) calculation for the HC–RR complex.

**S. no.**	**Energy components**	**Energy ± σ (kcal mol**^–^**^1^)**			
		**HC (a)**	**RR (b)**	**HC-RR (X)**	**Difference (X-a-b)**
1.	Bond	688.80 ± 22.97	8.64 ± 2.55	697.44 ± 23.20	0.00 ± 0.00
2.	Angle	1779.89 ± 33.64	24.14 ± 4.19	1804.33 ± 33.88	0.00 ± 0.00
3.	Dihedral	2820.38 ± 20.48	17.46 ± 2.61	2837.84 ± 20.75	0.00 ± 0.00
4.	VDWAALS	−1658.27 ± 25.37	−1.73 ± 2.15	−1685.64 ± −1685.64	−25.64 ± 4.33
5.	EEL	−15189.04 ± 157.73	−143.91 ± 12.72	−15369.50 ± −1685.63	−36.54 ± 33.36
6.	1–4 VDW	821.83 ± 12.12	5.81 ± 1.26	827.64 ± 12.20	0.00 ± 0.00
7.	1–4 EEL	10749.32 ± 43.44	21.373 ± 6.46	10770.69 ± 44.12	0.00 ± 0.00
8.	EGB	−4619.59 ± 135.84	−76.57 ± 10.11	−4647.11 ± 138.83	49.06 ± 32.77
9.	ESURF	96.94 ± 2.74	3.56 ± 0.167	96.63 ± 2.87	−3.87 ± 0.70
10.	G gas	12.90 ± 151.08	−67.93 ± 12.20	−117.19 ± 153.91	−62.17 ± 34.43
11.	G solv	−4522.65 ± 136.60	−73.01 ± 9.98	−4550.48 ± 139.60	45.18 ± 32.47
12.	G	−4509.74 ± 48.17	−140.94 ± 5.20	−4667.67 ± 48.98	−16.99 ± 5.99 *

*ΔG_*bind*_ (final estimated binding free energy, calculated as ΔG_*bind*_ = G_*HC–RR*_ − G_*HC*_ − G_*RR*_, where G_*HC–RR*_, G_*HC*_, and G_*RR*_ are the free energies of the HC–RR complex, HC protein, and RR ligand, respectively). VDWAALS, van der Waals contribution from MM; EEL, electrostatic energy; EGB, the electrostatic contribution to the solvation free energy; ESURF, non-polar contribution to the solvation free energy estimated by an empirical model; G gas, binding free energy at vacuum; G solv, binding free energy in solvated state.

## Discussion

### Rich Pool of Cyanobacterial OCAs Having Considerable Diversity and Sharing a Complex Phylogenetic Relationship

The distribution of putative OCAs among cyanobacteria exhibited a clear relation with larger genome size and morphological complexities (Kharwar et al., 2021), since the Oscillatoriales and Nostocales abundantly harbored them. This assumption was also supported by the absence of OCAs in the majority of the unicellular members of Synechococcales and their presence in the filamentous ones. However, the occurrence of considerable putative OCA genes among the Chroococcales, Pleurocapsales, and Chroococcidiopsidales suggested the existence of at least one more factor apart from genome size and morphology, favoring the prevalence of these genes. For example, the probable existence of OCA activity in colonies of *Microcystis* may confer advantages upon stress through altruistic adaptation, thereby enhancing inclusive fitness of the population (Durand et al., 2016). Further, the early origin of OCAs among cyanobacteria was depicted by the presence of these genes in the primitive cyanobacterium, *Gloeobacter* (Asplund-Samuelsson et al., 2012). Therefore, the loss of OCAs largely in the monophyletic clades of unicellular marine cyanobacteria *Prochlorococcus*, *Synechococcus*, and *Synechocystis* might be a cumulative consequence of extensive genome streamlining and solitary lifestyle (Ran et al., 2010; Larsson et al., 2011). On the other hand, the higher abundance of OCAs in the marine cyanobacterium *Trichodesmium erythraeum* IMS101 not only indicated the varied lifestyle preferences among cyanobacteria despite sharing the same habitat but also suggested lesser contribution of habitat in favoring the acquisition of these proteases. The occurrence of more OCAs in the mesophilic *Leptolyngbya* as compared to the thermophilic counterpart (*Thermoleptolyngbya* sp. PKUAC-SCTA183), further strengthened the previous assumption.

The rich pool of putative OCAs in cyanobacteria also displayed significant diversity based on the active site motif. While ∼72% accounted for wild-type OCAs (having conventional HC dyad) the rest ∼28% was pseudo-OCAs. In general, these pseudo-OCAs in cyanobacteria are considered as catalytically inert due to lack of functional HC motif in the active site, but in *Trypanosoma brucei* the role of TbMC4 (MCA having YS catalytic dyad) in the cell cycle progression and virulence warranted their activity in cellular processes (Proto et al., 2011). A recent *in silico* study also proposed the putative role of these pseudo-enzymes in photosynthesis (Klemenčič et al., 2019). Additionally, the presence of only true OCAs in *Gloeobacter* possibly indicated the origin of these pseudo-OCAs from their wild-type relatives in cyanobacteria (Klemenčič et al., 2019).

Being a rich repertoire of functional domains, apart from Peptidase C14, cyanobacterial OCAs presumably display multifunctionality, since these domains possess diverse catalytic capacities like protein–protein interactions (WD40), protein modification (FGE sulfatase), scaffold (HEAT_2), and signaling (GUN4). Mostly, these accessory domains are located C-terminally to Peptidase C14 but in some OCAs the C-terminally positioned Peptidase C14 domain was also observed, although these alterations in domain architecture may not have any functional amendments. In addition, the distribution of accessory domains was also uneven among cyanobacteria, since the OCAs from heterocytous strains with broader physiological abilities exhibited the presence of diverse accessory domains. The underlying reason is possibly the higher abundance of true OCAs in heterocytous strains. Additionally, the employment of these multidomain OCAs may benefit the physiologically robust life strategies. Furthermore, the presence of TM infers membrane localization of some OCAs, while others devoid of TM are probably cytosolic. The presence of putative cytosolic and membrane-bound true OCAs depicted the possibility of both intrinsic and extrinsic PCD in cyanobacteria (Bhattacharjee and Mishra, 2020), yet they are poorly appreciated in prokaryotes and require extensive research to validate.

MSA and phylogeny revealed significant sequence diversity and intermixing of cyanobacterial OCAs from different taxonomical orders. The wild-type OCAs were subsumed within nine distinct clades, whereas YS and YN motifs containing pseudo-variants formed two distinct clades, respectively; however, all other pseudo-OCAs were scattered throughout the tree. Mostly, the clades bracketed cyanobacterial strains with different morphological features and/or lifestyle preferences, suggesting the possibility of horizontal gene transfer (HGT) events for acquiring these genes (Bidle and Falkowski, 2004). Additionally, the appearance of several OCAs from the same strain in different clades may also indicate multiple HGT events (Bhattacharjee and Mishra, 2020). Interestingly, the YN pseudo-variant was only harbored by nitrogen-fixing multicellular cyanobacteria, whereas the YS variant was harbored by unicellular to multicellular strains, thus insinuating the possible physiological advantages of the YN variant in multicellular nitrogen-fixing strains. Besides, the distinct clades for YS and YN perhaps imply some functional similarities of these pseudo-proteases, while the scattered distribution of other pseudo-OCAs presumably implied their independent acquisition in cyanobacteria (Klemenčič et al., 2019). Nonetheless, in the future with more sequences of the putative cyanobacterial OCAs, a clearer phylogenetic relationship could be unveiled.

### Docking and MD Simulation Simultaneously Revealed Basic Substrate Specificity for Wild-Type OCA

The structural homology of three putative OCA variants with the HC, YS, and YN catalytic dyads is conferred by conserved CHF in their Peptidase C14 domain. Therefore, the substitution of catalytic motifs does not considerably amend the 3D conformation of these proteins (Klemenčič et al., 2019); however, the catalytic capabilities or substrate specificity will presumably alter. True OCAs preferably display substrate specificity for basic substrates due to critical negatively charged amino acid residues in their specificity pocket, as earlier observed in MaOC1 (Klemenčič et al., 2015). However, preference for basic substrates is common for all the characterized caspase homologs, till date. Expectedly, in our study, the putative wild-type OCA, WP_010999143.1 (HC), displayed high substrate affinity for the RR dipeptide, whereas no binding complex was formed with DEVD. Furthermore, the structural stability of the HC-RR complex was validated by analyzing the trajectory for the 100-ns MD simulation run. The negative binding energy calculated using MMGBSA also confirmed the binding efficacy of the RR dipeptide with the protease. The accommodation for the RR dipeptide was possibly determined by the two acidic residues, i.e., D132 and E176, in the binding pocket, thus providing Arg-P1 cleavage specificity to the protease. Similarly, in TbMC2 (MCA of *T. brucei*) D95 and D211 exhibited their significance in binding basic substrates (Bidle and Falkowski, 2004). Additionally, D353, E500, and D266 were previously identified as significant contributors for substrate-binding pockets in NtMC1 (MCA of *Nicotiana tabacum* L.), PCA MALT-1, and AtMC9 (MCA of *Arabidopsis thaliana*), respectively (Jong et al., 2011; Acosta-Maspons et al., 2014).

## Conclusion

The identification of caspase homologs in prokaryotes advocated for the possibility of a protease-based cell death mechanism among these life forms. However, the involvement of all these caspase homologs in cell execution pathways is still uncertain. The present study exclusively describes the distribution, diversity, abundance, domain architectures, and phylogenetic relationship of putative cyanobacterial OCAs. Further, this work unraveled the conservation of CHF and Arg-P1 specificity of wild-type OCA in *Nostoc* sp. PCC 7120 through detailed structural analysis, molecular docking, and MD simulation. Although the wild-type cyanobacterial OCAs possessed conserved fold and catalytic dyad, the protease activity and substrate specificity cannot be corroborated without experimental validations. However, delineating the functionality of the OCAs in cyanobacteria, progenitor of oxygenic photosynthesis, is crucial for understanding not only the evolution and acquisition of caspase homologs in the plant kingdom but also the physiological pertinence of these proteases in PCD.

## Data Availability Statement

The original contributions presented in the study are included in the article/Supplementary Material, further inquiries can be directed to the corresponding author.

## Author Contributions

SB conceptualized the study and performed the computational analysis. SB and SK retrieved sequences from the database and performed the curation and analysis. AKM conceived and supervised the work. SB wrote the manuscript with contribution and thorough revision from SK and AKM. All authors contributed to the article and approved the submitted version.

## Conflict of Interest

The authors declare that the research was conducted in the absence of any commercial or financial relationships that could be construed as a potential conflict of interest.

## Footnotes

1 https://www.ncbi.nlm.nih.gov/

2 https://www.ncbi.nlm.nih.gov/Structure/bwrpsb/bwrpsb.cgi

3 http://www.cbs.dtu.dk/services/TMHMM-2.0/

4 http://skylign.org/

5 https://swissmodel.expasy.org/interactive

6 https://zhanglab.ccmb.med.umich.edu/ModRefiner/

7 https://servicesn.mbi.ucla.edu/PROCHECK/

8 https://www.reading.ac.uk/bioinf/ModFOLD/

9 https://www.ebi.ac.uk/thornton-srv/databases/cgi-bin/pdbsum/GetPage.pl?pdbcode=index.html

## Supplementary Material

Supplementary Figure 1 Neighbor-joining tree defining phylogenetic relationship within distinct **(A)** HC I, **(B)** HC II and **(C)** HC III clades.

Supplementary Figure 2 Neighbor-joining tree defining phylogenetic relationship within distinct HC IV clades.

Supplementary Figure 3 Neighbor-joining tree defining phylogenetic relationship within distinct **(A)** HC V, **(B)** HC VI, **(C)** HC VII, **(D)** HC VIII and **(E)** HC IX clades.

Supplementary Figure 4 Neighbor-joining tree defining phylogenetic relationship within distinct **(A)** YS and **(B)** YN clades.

Supplementary Table 1 Domain positioning and configuration of all the putative cyanobacterial orthocaspases.

Supplementary Table 2 Detail of templates used for model refinement and energy minimization for three homology models of putative orthocaspases of *Nostoc* sp. PCC 7120.

Supplementary Table 3 Structure quality assessment using Ramachandran plot (PROCHECK) and ModFold8.

Supplementary Table 4 Detailed description of cyanobacterial strains and the number of diverse putative orthocaspases harbored by them.

## Abbreviations

DELTA-BLAST: domain enhanced lookup time accelerated-BLAST
DEVD: aspartyl-glutamyl-valyl-aspartate
MMGBSA: molecular mechanics/generalized born surface area
OCA: orthocaspase
PCD: programmed cell death
RMSD: root mean square deviation
RMSF: root mean square fluctuations
RR: arginylarginine.
