# Supplementary figures and images for: Insights Into the Phylogenetic Distribution, Diversity, Structural Attributes, and Substrate Specificity of Putative Cyanobacterial Orthocaspases
Source: Front Microbiol. 2021 Jul 2;12:682306. doi: 10.3389/fmicb.2021.682306 (PMC8283722; doi:10.3389/fmicb.2021.682306)

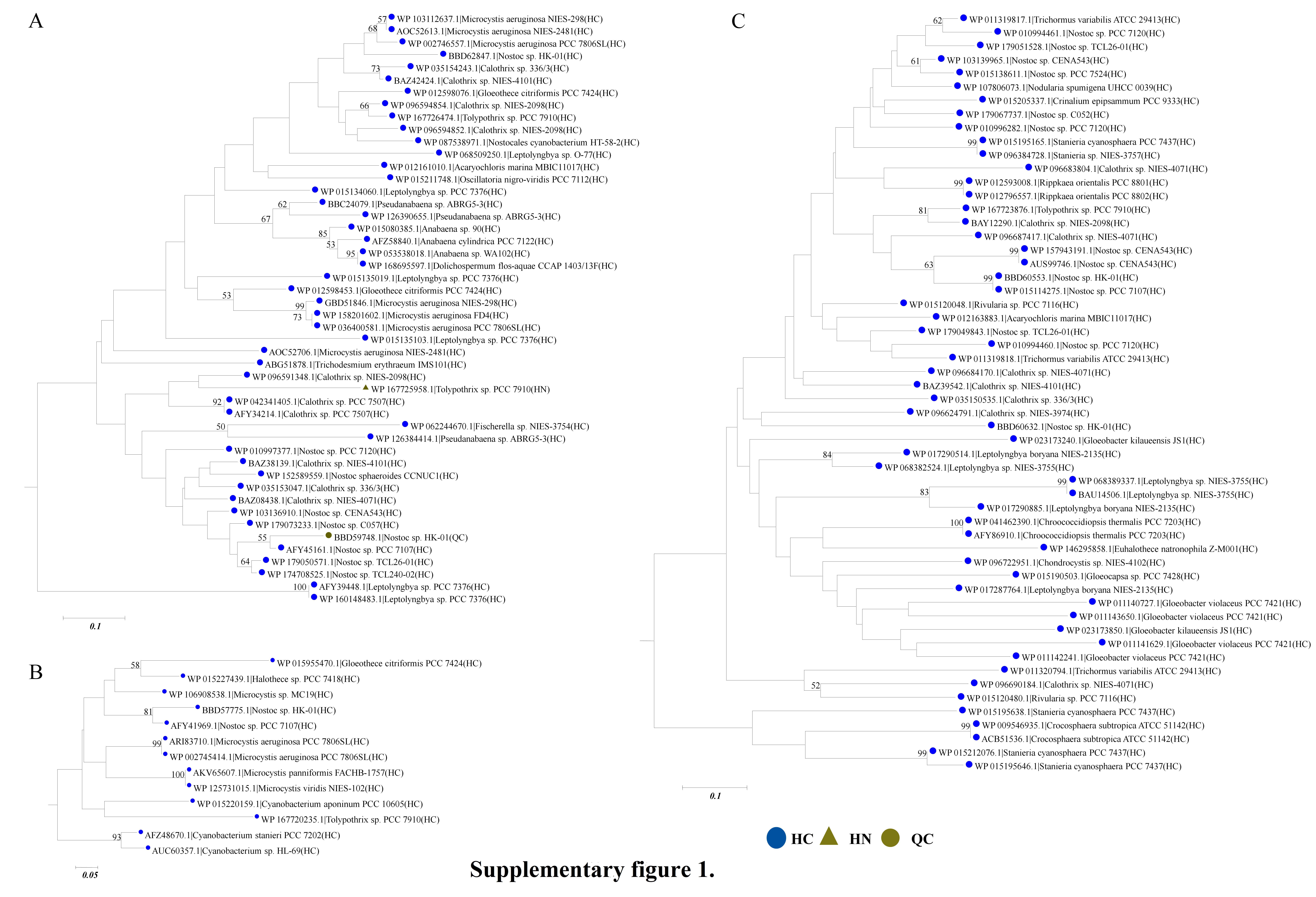

Supplement: Supplementary Figure 1 — Neighbor-joining tree defining phylogenetic relationship within distinct (A) HC I, (B) HC II and (C) HC III clades. [file Image_1.TIF]

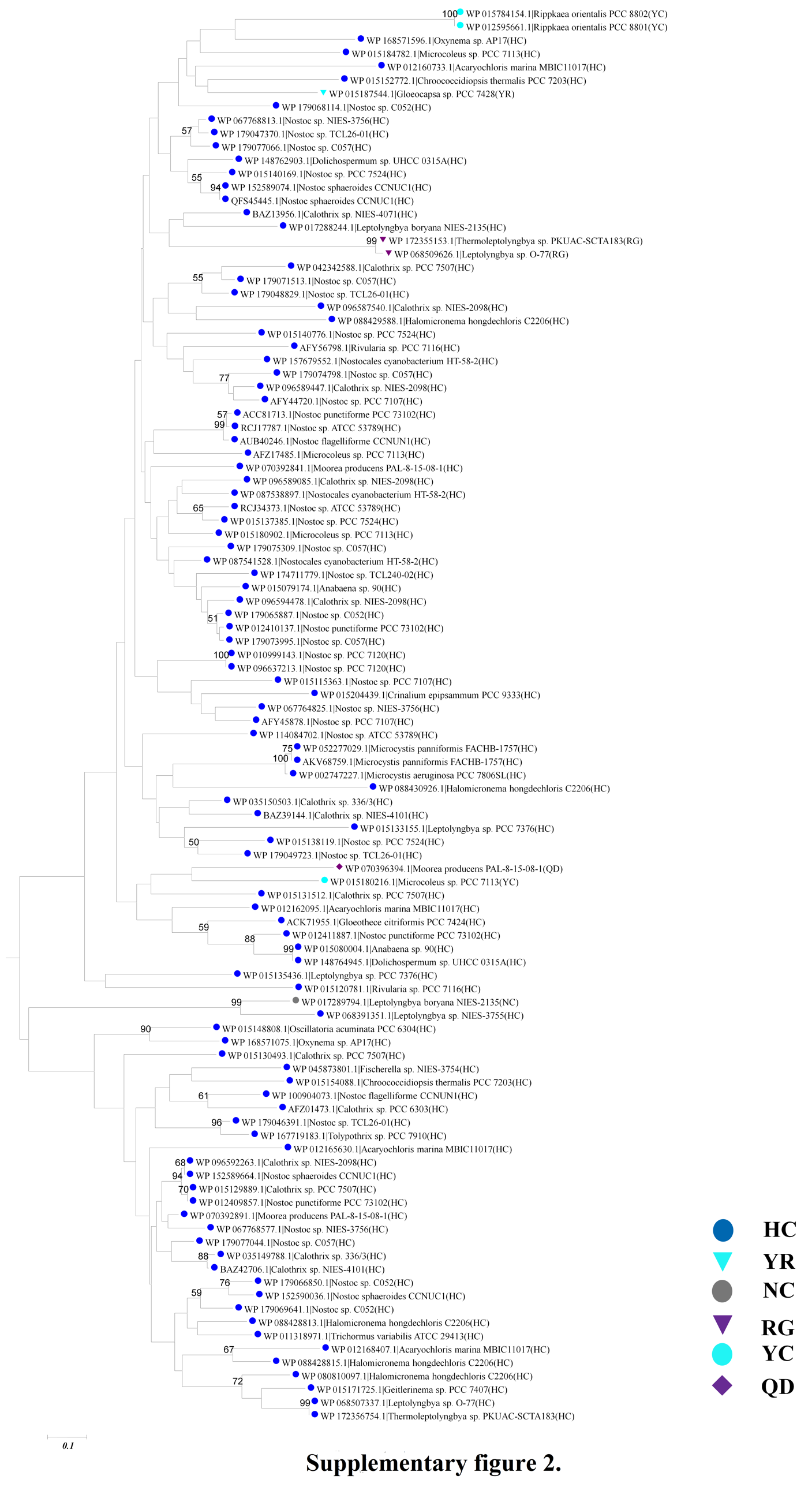

Supplement: Supplementary Figure 2 — Neighbor-joining tree defining phylogenetic relationship within distinct HC IV clades. [file Image_2.TIF]

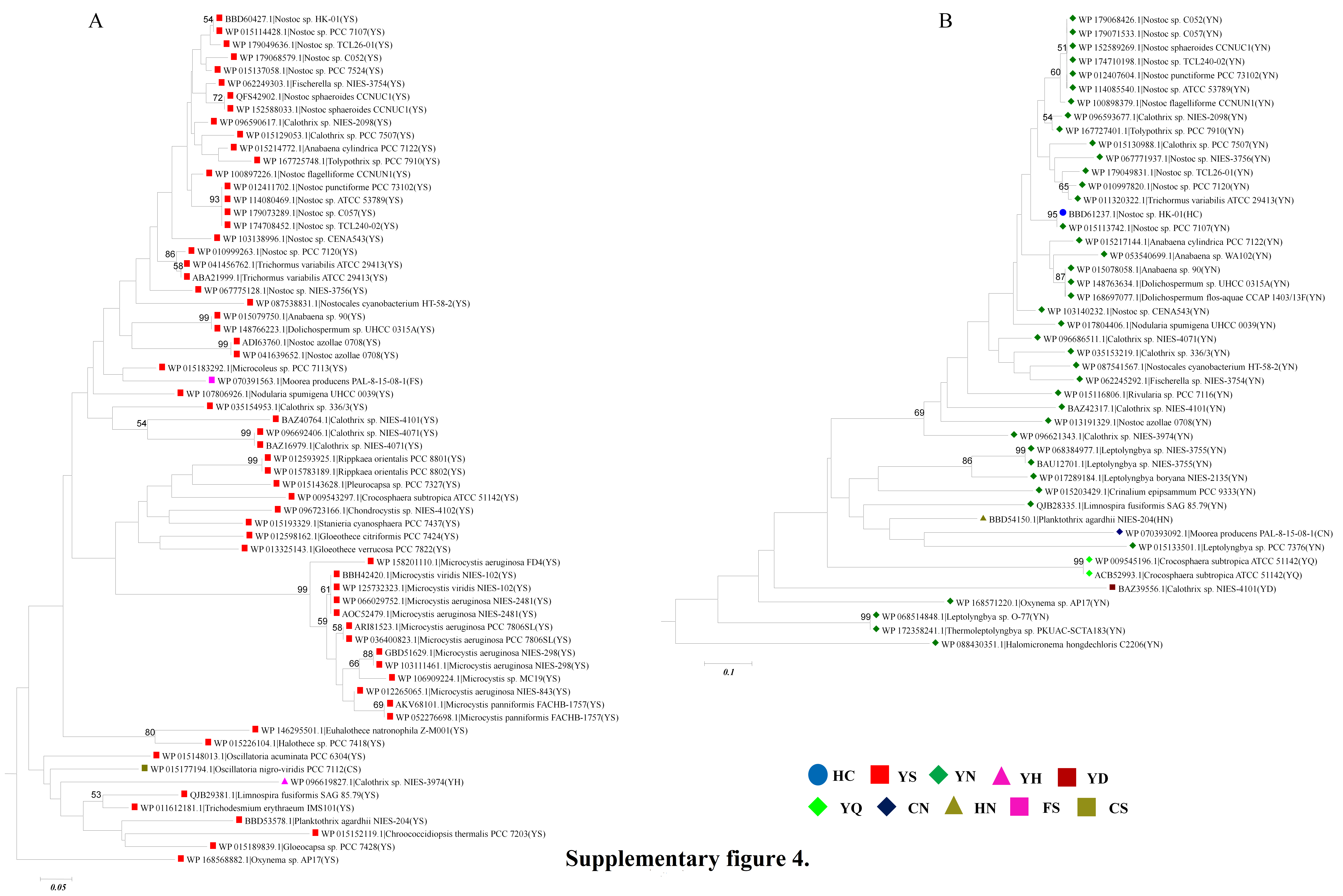

Supplement: Supplementary Figure 4 — Neighbor-joining tree defining phylogenetic relationship within distinct (A) YS and (B) YN clades. [file Image_4.TIF]
