# Supplementary material for: Insights Into the Phylogenetic Distribution, Diversity, Structural Attributes, and Substrate Specificity of Putative Cyanobacterial Orthocaspases
Source: Front Microbiol. 2021 Jul 2;12:682306. doi: 10.3389/fmicb.2021.682306 (PMC8283722; doi:10.3389/fmicb.2021.682306)
Supplement: Supplementary Table 1 — Domain positioning and configuration of all the putative cyanobacterial orthocaspases. [file Table_1.pdf]

Supplementary Table 1: Domain positioning and configuration of all the putative cyanobacterial orthocaspases.

S.no.	Organism	NCBI accession no.	Size (a.a.)	TM	Domains	Domain position (a.a.)	Catalytic dyad
1	*Acaryochloris marina* MBIC11017	WP_012162095.1	595		Peptidase C14	8-220	HC
		WP_012161010.1	633		Peptidase C14	5-224	HC
					FGE-sulfatase	383-626	
		WP_012164848.1	767		Peptidase C14	44-308	YG
		WP_012165630.1	1013	749-771	Peptidase C14	5-230	HC
		WP_012168479.1	1007		Peptidase C14	1-202	HC
					DEAD	284-446	
					Helicase_C	578-660	
		WP_012163883.1	1703		Peptidase C14	23-231	HC
					AAA_16	550-695	
					WD40	1473-1511, 1594-1631, 1554-1591,1112-1143, 1352-1389, 1229-1265,1515-1550, 1269-1306, 1398-1425	
		WP_012168407.1	1037		Peptidase C14	5-235	HC
					AAA_16	282-403	
					Pentapeptide	920-869, 865-904, 830-869, 805-844, 940-980	
		WP_0121607331.1	1044		Peptidase C14	231-425	HC
					WD40	836-874, 962-1000, 878-916, 920-958, 794-832, 1004-1042	
2	*Anabaena cylindrica* PCC 7122	WP_015215428.1	399		Peptidase C14*	45-201	HC
		AFZ58840	341		Peptidase C14	6-242	HC
		WP_015214772.1	727		Peptidase C14	42-292	YS
		WP_015217806.1	2249		Peptidase C14*	3-161	HC
		WP_015217144.1	618	352-371	Peptidase C14	5-208	YN
3	*Anabaena* sp. 90	WP_015081610.1	1154		Peptidase C14	6-277	HC
		WP_015080004.1	725		Peptidase C14	4-215	HC
					FGE-sulfatase	476-717	
		WP_015080385.1	622		Peptidase C14	6-238	HC
		WP_015079174.1	838		Peptidase C14	3-232	HC
		WP_015079750.1	714		Peptidase C14	42-298	YS
		WP_015078058.1	615	351-370	Peptidase C14	5-228	YN
		WP_015081248.1	328		Peptidase C14	8-260	HC
4	*Anabaena* sp. WA102	WP_053539780.1	758		Peptidase C14	45-316	HC
					DUF4384	592-678	
		WP_053540514.1	709		Peptidase C14*	42-145	YS
		WP_053538018.1	626		Peptidase C14	6-242	HC
		WP_053540699.1	615	351-370	Peptidase C14	5-213	YN
5	*Calothrix* sp. 336/3	WP_035150622.1	745		Peptidase C14	42-289	HC
					DUF4384	584-668	
		WP_035149616.1	1148		Peptidase C14	5-275	HC
		WP_046814694.1	1463		Peptidase C14*	23-216	HC
					AAA_16	322-358	
					WD40	1120-1158	
		WP_035150503.1	764		Peptidase C14	4-230	HC
					FGE-sulfatase	516-757	
		WP_035153047.1	657		Peptidase C14	5-231	HC
					FGE-sulfatase	416-652	
		WP_052754496.1	783		Peptidase C14	56-288	HC
					Sel1	345-379, 502-537	
					DUF 2610	701-777	
		WP_035154953.1	720		Peptidase C14	42-258	YS
		WP_035154243.1	768		Peptidase C14	5-240	HC
					FGE-sulfatase	520-760	
		WP_035156535.1	1695		Peptidase C14	25-238	HC
					WD40	1446-1483, 1230-1267, 1273-1370, 1530-1567, 1489-1525, 1582-1616, 1104-1141, 1357-1393, 1146-1183, 1314-1351, 1071-1100, 1407-1441, 1619-1654	
		WP_035149788.1	1012	689-720	Peptidase C14	5-201	HC
					Pentapeptide	856-910, 786-825, 811-849, 946-980, 901-960	
		WP_168163431.1	1354		Peptidase C14	1039-1290	HC
					HEAT_2	700-783, 800-886, 575-660, 524-598	
		WP_035153219.1	666		Peptidase C14	5-207	YN
6	*Calothrix* sp. NIES-2098	WP_096591348.1	503		Peptidase C14	5-231	HC
					GUN4	345-468	
		WP_096589085.1	706		Peptidase C14	3-233	HC
					FGE-sulfatase	455-697	
		WP_096594478.1	841	379-401	Peptidase C14	3-232	HC
					Peripla BP_6	493-827	
		BAY09269.1	146		Peptidase C14*	5-106	H-
		WP_096589447.1	526		Peptidase C14	3-235	HC
					GUN4	369-523	

		WP_096587540.1	552		Peptidase C14	3-237	HC
					GUN4	393-528	
		WP_096590617.1	703		Peptidase C14	42-302	YS
		WP_096587679.1	995		Peptidase C14	4-222	HC
					DEAD	282-444	
					Helicase_C	581-658	
		WP_096587043.1	346		Peptidase C14	9-201	HC
		WP_096593677.1	652	374-393	Peptidase C14	5-228	YN
		WP_096588888.1	1715		Peptidase C14*	25-221	HC
					AAA_16	549-682	
					WD40	1269-1307, 1477-1513, 1519-1555, 1601-1639, 1187-1224, 1107-1144, 1228-1265, 1356-1392, 1643-1681, 1310-1348, 1559-1597, 1434-1472	
		WP_096594854.1	610		Peptidase C14	5-210	HC
					FGE-sulfatase	364-602	
		WP_096594852.1	634		Peptidase C14	5-194	HC
		WP_096592263.1	952	702-724	Peptidase C14	5-198	HC
					AAA_16	273-396	
					Pentapeptide	767-805, 791-835, 831-870, 856-896, 881-919	
		BAY12290.1	742		Peptidase C14	25-227	HC
					GUN4	550-696	
7	*Calothrix* sp. NIES-3974	WP_096624791.1	1715		Peptidase C14	23-227	HC
					AAA_16	538-690	
					WD40	1129-1166, 1632-1670, 1509-1546, 1590-1627, 1344-1381, 1549-1587, 1426-1463, 1303-1340, 1390-1423, 1094-1125, 1170-1208	
		WP_096619827.1	718		Peptidase C14	43-286	YH
		WP_096621343.1	659	342-364	Peptidase C14	5-206	YN
		WP_096621031.1	785	753-775	Peptidase C14*	12-147	YC
					AAA_16	277-141	
8	*Calothrix* sp. NIES-4071	WP_096688233.1	749		Peptidase C14	45-314	HC
					DUF4384	585-670	
		WP_096689425.1	753		Peptidase C14	45-325	HC
					DUF4384	593-671	
		WP_096684453.1	1505		Peptidase C14	8-256	HC
					WD40	1136-1173, 1057-1091, 1386-1422, 1347-1382, 1426-1463, 1305-1343, 1094-1131, 1268-1326, 1217-1253	
		BAZ08438.1	649		Peptidase C14	4-241	HC
		BAZ13956.1	530		Peptidase C14	3-232	HC
					GUN4	376-511	
		WP_096684452.1	690		Peptidase C14	6-279	HC
		WP_096684104.1	1269		Peptidase C14	123-397	HC
		BAZ08283.1	187		Peptidase C14*	8-150	HC
		WP_096692406.1	705		Peptidase C14	42-280	YS
		BAZ16979.1	706		Peptidase C14	43-281	YS
		WP_096690184.1	698		Peptidase C14	25-229	HC
		WP_096687417.1	708		Peptidase C14	25-224	HC
					GUN4	533-680	
		WP_096684170.1	1648		Peptidase C14	22-219	HC
					AAA_16	515-671	
					WD40	1081-1115, 1119-1157, 1490-1527, 1532-1570, 1244-1282, 1043-1075, 1160-1198, 1202-1240, 1285-1322, 1573-1610, 1457-1485	
		WP_096686511.1	681	379-401	Peptidase C14	5-206	YN
		WP_096683804.1	1658		Peptidase C14	23-210	HC
					WD40	1145-1183, 1422-1459, 1225-1263, 1064-1100, 1464-1500, 1304-1341, 1381-1418, 1189-1220, 1587-1624	
9	*Calothrix* sp. NIES-4101	BAZ40681.1	746		Peptidase C14	42-271	HC
					DUF4384	584-668	
		BAZ42600.1	300		Peptidase C14	42-292	HC
		BAZ39144.1	749		Peptidase C14	4-233	HC
					FGE-sulfatase	500-742	
		BAZ37666.1	1155		Peptidase C14	5-282	HC
		BAZ37671.1	337		Peptidase C14	10-276	HC
		BAZ38139.1	619		Peptidase C14	5-239	HC
					FGE-sulfatase	380-615	
		BAZ40764.1	730		Peptidase C14	42-286	YS
		BAZ42706.1	246		Peptidase C14	5-201	HC

		BAZ37947.1	565		Peptidase C14	250-499	HC
					HEAT_2	10-95, 72-181, 137-207	
		BAZ42424.1	660		Peptidase C14	5-240	HC
					FGE-sulfatase	379-629	
		BAZ38659.1	732		Peptidase C14*	23-227	HC
		BAZ39542.1	1663		Peptidase C14	23-216	HC
					AAA_16	528-701	
					WD40	1550-1588, 1144-1181, 1185-1223, 1063-1100, 1508-1545, 1382-1420, 1103-1140, 1466-1504, 1435-1461, 1226-1264, 1304-1344, 1592-1630	
		BAZ42317.1	711	370-392	Peptidase C14	5-207	YN
		BAZ37670.1	1510		Peptidase C14	10-280	HC
					WD40	1135-1173, 1095-1132, 1389-1427, 849-881, 1266-1298, 935-964, 1180-1214, 884-992, 1357-1386, 981-1010, 1219-1255, 1013-1651, 1309-1342, 1430-1472	
		BAZ39556.1	1263		Peptidase C14	701-944	YD
					FGE-sulfatase	1000-1255	
10	*Calothrix* sp. PCC 6303	AFZ01473.1	580		Peptidase C14	5-247	HC
					TPR 19	425-480	
11	*Calothrix* sp. PCC 7507	WP_015127455.1	746		Peptidase C14	42-307	HC
					DUF4384	584-668	
		WP_042341405.1	492		Peptidase C14	5-229	HC
					GUN4	332-456	
		AFY34214.1	520		Peptidase C14	33-257	HC
					GUN4	360-484	
		WP_015129889.1	1102		Peptidase C14	5-239	HC
					AAA_16	273-394	
					Pentapeptide	861-899, 901-940, 836-875, 771-809, 791-829, 966-994	
					Pentapeptide_4	987-1064	
		WP_015131512.1	543		Peptidase C14	4-214	HC
					WD40	290-328, 504-541, 248-286, 426-458, 462-500, 342-374	
		WP_042342588.1	351		Peptidase C14	3-233	HC
		WP_015130988.1	659	382-401	Peptidase C14	5-206	YN
		WP_015129053.1	723		Peptidase C14	49-300	YS
		WP_015131812.1	1713		Peptidase C14*	25-221	HC
					AAA_16	551-680	
					WD40	1178-1215, 1474-1511, 1599-1637, 1516-1553, 1099-1134, 1557-1594, 1641-1679, 1137-1175, 1351-1386, 1302-1340, 1436-1469	
		WP_015130493.1	1782		Peptidase C14	5-244	HC
					AAA_16	279-425	
					WD40	926-964, 1464-1502, 1339-1375, 1167-1200, 967-1005, 886-923, 1262-1290, 1066-1092	
12	*Chondrocystis* sp. NIES-4102	WP_096723166.1	764		Peptidase C14	53-316	YS
					DUF4384	599-690	
		WP_096722951.1	1589		Peptidase C14	23-224	HC
					AAA_16	543-665	
					WD40	1497-1534, 1409-1447, 1450-1487, 1367-1405, 1326-1363, 1236-1279, 1538-1570	
13	*Chroococcidiopsis thermalis* PCC 7203	WP_015152119.1	785		Peptidase C14	42-328	YS
		WP_041462390.1	719		Peptidase C14	23-230	HC
		AFY86910.1	737		Peptidase C14	41-248	HC
		WP_015152772.1	641		Peptidase C14	3-223	HC
					WD40	435-473, 519-557, 477-515, 561-599, 392-431, 359-388, 603-639	
		WP_015154088.1	1464		Peptidase C14	9-199	HC
					AAA_22	288-427	
					MAP7	628-730	
					WD40	1162-1194, 1365-1403, 1246-1278, 1288-1320, 1078-1110, 1330-1362, 910-942, 868-900, 1204-1236, 1036-1068, 952-984, 994-1026, 1118-1152	

14	*Crinalium epipsammum* PCC 9333	WP_015204439.1	512		Peptidase C14	3-235	HC
		WP_015201768.1	1373	527-546, 746-768	Peptidase C14	8-268	HC
					AAA_16	310-464	
					WD40	1214-1251, 915-953, 997-1035, 1132-1170, 956-994, 1173-1210, 1260-1293, 839-871, 1296-1334, 874-912	
		WP_015201767.1	1333	1266-1288	Peptidase C14	6-298	HC
		WP_015205337.1	1759		Peptidase C14	25-229	HC
					AAA_16	566-704	
					WD40	1470-1508, 1554-1592, 1305-1342, 1346-1384, 1387-1424, 1178-1216, 1220-1257, 1596-1663, 1262-1299, 1143-1175, 1640-1675, 1679-1717	
		WP_015203429.1	594	329-351	Peptidase C14	6-224	YN
15	*Crocosphaera subtropica* ATCC 51142	WP_009543297.1	737		Peptidase C14	45-305	YS
		WP_009546935.1	1748		Peptidase C14	21-236	HC
					AAA_16	541-575	
					WD40	1511-1549, 1552-1590, 1184-1222, 1470-1508, 1593-1631, 1267-1305, 1672-1710, 1225-1264, 1641-1669, 1308-1344, 1134-1181, 1348-1390	
		ACB51536.1	1750		Peptidase C14	23-238	HC
					AAA_16	543-577	
					WD40	1513-1551, 1554-1592, 1189-1224, 1472-1510, 1595-1633, 1269-1307, 1674-1712, 1227-1266, 1643-1671, 1310-1346, 1136-1183, 1350-1392	
		WP_009545196.1	562		Peptidase C14	5-195	YQ
		ACB52993.1	564		Peptidase C14	7-197	YQ
16	*Cyanobacterium aponinum* PCC 10605	WP_015220159.1	414	5-27	Peptidase C14	74-296	HC
		WP_015221282.1	278		Peptidase C14	4-270	HC
		WP_150108998.1	711		Peptidase C14*	41-149	YL
		AFZ54050.1	712		Peptidase C14*	42-150	YL
17	*Cyanobacterium* sp. HL-69	AUC60357.1	482		Peptidase C14	61-282	HC
					DUF2808	337-478	
		AUC61872.1	712		Peptidase C14*	47-130	Y-
18	*Cyanobacterium stanieri* PCC 7202	AFZ48670.1	482	7-29	Peptidase C14	61-282	HC
					DUF2808	336-477	
		AFZ48265.1	713		Peptidase C14*	47-130	Y-
19	*Cyanothece* sp. PCC 7425	WP_049770902.1	352		Peptidase C14	42-298	YG
20	*Dactylococcopsis salina* PCC 8305	WP_041235413.1	128		Peptidase C14*	51-128	_
21	*Dolichospermum flos-aquae* CCAP 1403/13F	WP_168694996.1	765		Peptidase C14	45-360	HC
		WP_168695385.1	714		Peptidase C14*	42-154	Y-
		WP_168695597.1	626		Peptidase C14	6-242	HC
		WP_168697077.1	615	351-370	Peptidase C14	5-228	YN
22	*Dolichospermum* sp. UHCC 0315A	WP_148762903.1	542		Peptidase C14	3-235	HC
		WP_148764945.1	718		Peptidase C14	4-215	HC
					FGE-sulfatase	476-710	
		WP_148766223.1	714		Peptidase C14	42-298	YS
		WP_148763634.1	615	351-370	Peptidase C14	5-228	YN
23	*Euhalothece natronophila* Z-M001	WP_146295501.1	752		Peptidase C14	47-312	YS
		WP_146295858.1	1670		Peptidase C14	25-224	HC
					WD40	1399-1436, 1561-1599, 1439-1477, 1078-1110, 1113-1151, 1521-1559, 1480-1518, 1194-1233, 1367-1395, 1325-1358	
24	*Fischerella* sp. NIES-3754	WP_062244670.1	544		Peptidase C14	5-229	HC
					GUN4	387-527	
		WP_062249303.1	731		Peptidase C14	42-292	YS
		WP_052335393.1	986		Peptidase C14*	3-153	HC
					AAA_16	290-445	
					Trypsin_2	797-939	
		WP_052335392.1	1348		Peptidase C14*	5-201	HC
					AAA_16	278-434	
					WD40	1043-1071	
		WP_045873801.1	1707		Peptidase C14	5-205	HC
		WP_062245292.1	614	328-347	Peptidase C14	5-202	YN
25	*Geitlerinema* sp. PCC 7407	WP_015170551.1	724		Peptidase C14	42-324	HC
					DUF4384	569-654	
		WP_015171787.1	735		Peptidase C14*	42-196	HC
		WP_015171588.1	1121		Peptidase C14	3-227	HC
					WD40	825-863	
		WP_015171725.1	1619		Peptidase C14	5-225	HC
					AAA_16	270-417	

					WD40	1393-1431, 1443-1470, 990-1019	
		WP_015171320.1	790		Peptidase C14*	45-135	Y-
					DUF4384	623-717	
26	*Geminocystis* sp. NIES-3708	WP_066345536.1	717		Peptidase C14*	47-183	Y-
27	*Geminocystis* sp. NIES-3709	WP_066116041.1	712		Peptidase C14*	45-179	Y-
		BAQ63796.1	665		Peptidase C14*	2-132	Y-
28	*Gloeobacter kilaueensis* JS1	WP_023174686.1	1730		Peptidase C14*	25-222	HC
					WD40	1287-1325, 1123-1160, 1328-1365, 1410-1448, 1164-1201, 1611-1649, 1451-1488, 1570-1608, 1653-1689	
		WP_023173240.1	1659		Peptidase C14	22-209	HC
					AAA_16	510-660	
					WD40	1169-1206, 1251-1288, 1210-1247, 1128-1165, 1052-1084, 1456-1493, 1087-1125, 1414-1452, 1293-1330, 1539-1574, 1496-1534, 1335-1370	
		WP_023173850.1	1707		Peptidase C14	28-227	HC
					AAA_16	542-664	
					WD40	1164-1202, 1123-1161, 1292-1328, 1248-1285, 1541-1578, 1584-1621, 1206-1244, 1460-1497, 1332-1370	
		WP_023174562.1	401		Peptidase C14	17-292	HC
29	*Gloeobacter violaceus* PCC 7421	WP_164928426.1	717		Peptidase C14	5-292	HC
		BAC88035.1	719		Peptidase C14	7-294	HC
		WP_011140727.1	1671		Peptidase C14	20-222	HC
					AAA_16	531-702	
					WD40	1133-1171, 1215-1253, 1543-1580, 1502-1539, 1297-1334, 1584-1621, 1256-1294, 1092-1129, 1051-1088, 1338-1376, 1419-1459	
		WP_011141629.1	1730		Peptidase C14	25-223	HC
					AAA_16	539-660	
					WD40	1613-1651, 1572-1609, 1246-1284, 1288-1326, 1329-1366, 1123-1161, 1629-1689, 1411-1449, 1531-1568, 1164-1201	
		WP_011143650.1	1682		Peptidase C14	25-230	HC
					AAA_16	530-658	
					WD40	1290-1324, 1613-1651, 1410-1447, 1328-1365, 1317-1407, 1205-1242, 1087-1115, 1165-1202, 1451-1487	
		WP_011142241.1	1721		Peptidase C14	24-227	HC
					AAA_16	546-668	
					WD40	1214-1251, 1296-1334, 1255-1292, 1097-1129, 1418-1456, 1173-1210, 1337-1374, 1500-1538, 1381-1414, 1541-1579, 1132-1169	
30	*Gloeocapsa* sp. PCC 7428	WP_015189839.1	755		Peptidase C14	42-320	YS
		WP_015190503.1	1693		Peptidase C14	22-227	HC
					AAA_16	548-691	
					WD40	1579-1617, 1286-1324, 1456-1493, 1538-1575, 1497-1535, 1093-1125, 1128-1165, 1246-1283, 1206-1243, 1327-1363, 1168-1203	
		WP_015187544.1	605		Peptidase C14	3-200	YR
					WD40	357-395, 525-563, 483-521, 567-604	
31	*Gloeothece citriformis* PCC 7424	WP_015955470.1	490		Peptidase C14	56-285	HC
		WP_012598162.1	789		Peptidase C14	53-327	YS
		ACK71955.1	925		Peptidase C14	4-214	HC
					FGE-sulfatase	677-919	
		WP_015955555.1	902		Peptidase C14	3-213	HC
					GGDEF	734-895	
		WP_012598453.1	670		Peptidase C14	4-244	HC
					GUN4	510-642	
		WP_012598076.1	654		Peptidase C14	5-223	HC

					FGE-sulfatase	393-648	
32	*Gloeothece verrucosa* PCC 7822	WP_013325143.1	814		Peptidase C14	53-323	YS
		WP_013322613.1	1444		Peptidase C14	3-213	HC
					WD40	1022-1059, 1274-1311, 1106-1143, 1190-1227, 896-933, 980-1017, 855-891, 1358-1394,939-975, 1316-1353, 1065-1101, 1233-1269, 1149-1185, 820-849	
		WP_013321028.1	346		Peptidase C14	8-236	HC
		WP_049802848.1	860		Peptidase C14*	5-152	HC
					AAA_16	281-400	
33	*Halomicronema hongdechloris* C2206	WP_080812381.1	720		Peptidase C14	45-313	HC
					DUF4384	568-654	
		WP_080805631.1	768		Peptidase C14*	42-129	H-
		ASC73447.1	739		Peptidase C14	42-314	HC
					DUF4384	575-661	
		WP_080811483.1	746		Peptidase C14	49-321	HC
					DUF4384	582-668	
		WP_088428813.1	1138		Peptidase C14	5-196	HC
					AAA_16	275-429	
					Pentapeptide	807-846, 977-1015, 832-871, 987-1056, 872-959	
		WP_088428815.1	958		Peptidase C14	5-232	HC
					AAA_16	278-399	
					Pentapeptide	818-855, 838-875, 795-825	
		WP_088529588.1	664		Peptidase C14	3-232	HC
		WP_080810097.1	843		Peptidase C14	5-230	HC
					AAA_16	281-407	
		WP_088430926.1	657		Peptidase C14	4-239	HC
					FGE-sulfatase	423-649	
		WP_088430464.1	760		Peptidase C14	44-305	YG
		WP_080807484.1	348		Peptidase C14	8-231	HC
		WP_088430351.1	566		Peptidase C14	5-224	YN
34	*Halothece* sp. PCC 7418	WP_015227439.1	598		Peptidase C14	41-254	HC
					DUF2808	451-592	
		WP_015226104.1	752		Peptidase C14	51-314	YS
35	*Leptolyngbya boryana* NIES-2135	WP_017292124.1	674		Peptidase C14	34-308	HC
		WP_017286212.1	688		Peptidase C14	32-305	HC
		WP_017290511.1	695		Peptidase C14	35-292	YG
		WP_017288244.1	656		Peptidase C14	3-225	HC
					FGE-sulfatase	412-649	
		WP_017287764.1	821		Peptidase C14	23-229	HC
		WP_017290514.1	1634		Peptidase C14	21-222	HC
					AAA_16	515-688	
					WD40	1130-1168, 1254-1292, 1172-1210, 1092-1127, 1449-1486, 1530-1565, 1295-1332, 1408-1446, 1371-1404, 1054-1086, 1490-1525, 1217-1250	
		WP_017290885.1	1087		Peptidase C14	18-220	HC
					WD40	987-1023, 849-886, 811-845, 948-975, 891-928	
		WP_017289184.1	489	346-368	Peptidase C14	5-210	YN
		WP_017289794.1	588	427-449	Peptidase C14	4-193	NC
					TPR_1	500-532	
					TPR_16	504-564	
		WP_017292148.1	2215		Peptidase C14	28-232	HC
		BAY59491.1	2216		Peptidase C14	29-233	HC
36	*Leptolyngbya* sp. NIES-3755	WP_068381839.1	209		Peptidase C14*	43-206	HC
		WP_068394534.1	683		Peptidase C14	34-308	HC
		WP_068392066.1	700		Peptidase C14*	38-122	F-
		WP_068384977.1	531	384-406	Peptidase C14	18-225	YN
		BAU12701.1	518	371-393	Peptidase C14	5-212	YN
		WP_068382524.1	1612		Peptidase C14	24-221	HC
					AAA_16	508-679	
					WD40	1226-1264, 1061-1099, 1508-1545, 1384-1422, 1102-1139, 1346-1380, 1267-1304, 1425-1462, 1026-1058, 1469-1503, 1144-1181	
		WP_068389337.1	1105		Peptidase C14	30-231	HC
					WD40	1004-1040, 826-861, 865-903, 907-944, 965-991	
		BAU14506.1	1088		Peptidase C14	13-214	HC
					WD40	987-1023, 812-844, 848-886, 890-927, 948-974	
		WP_068391351.1	580	421-443	Peptidase C14	4-193	HC
					TPR_1	494-525	
					TPR_16	498-560, 460-526	

37	*Leptolyngbya* sp. O-77	WP_068514848.1	386		Peptidase C14	5-216	YN
		WP_068507337.1	1804		Peptidase C14	5-195	HC
					AAA_16	273-411	
					WD40	1653-1691, 836-867, 1562-1608, 1620-1647, 1059-1087	
		WP_068513554.1	755		Peptidase C14	44-308	LG
		WP_068509250.1	558		Peptidase C14	5-220	HC
		WP_068509626.1	1209		Peptidase C14	3-222	RG
38	*Leptolyngbya* sp. PCC 7376	WP_015136072.1	283		Peptidase C14	14-283	HC
		WP_015135019.1	573		Peptidase C14	4-225	HC
					FGE-sulfatase	354-569	
		WP_015132258.1	680		Peptidase C14*	44-143	Y-
		WP_015133155.1	654		Peptidase C14	3-204	HC
					FGE-sulfatase	399-650	
		AFY39448.1	640		Peptidase C14	5-226	HC
		WP_160148483.1	607		Peptidase C14	8-229	HC
		WP_015135436.1	712		Peptidase C14	4-231	HC
					FGE-sulfatase	461-709	
		WP_015134060.1	518		Peptidase C14	5-232	HC
		WP_015135103.1	640		Peptidase C14	5-199	HC
					FGE-sulfatase	397-633	
		WP_015133501.1	887	374-396	Peptidase C14	9-215	YN
39	*Limnospira fusiformis* SAG 85.79	QJB27797.1	398		Peptidase C14	45-305	HC
		QJB29381.1	782		Peptidase C14	44-313	YS
		QJB26851.1	348		Peptidase C14	8-236	HC
		QJB26854.1	348		Peptidase C14	8-250	HC
		QJB28335.1	624	343-360	Peptidase C14	5-207	YN
40	*Microcoleus* sp. PCC 7113	WP_015185307.1	385		Peptidase C14*	31-244	HC
		WP_015183451.1	742		Peptidase C14	45-302	HC
					DUF4384	581-667	
		WP_015185692.1	743		Peptidase C14	42-326	HC
					DUF4384	580-662	
		WP_015180542.1	397		Peptidase C14	45-308	HC
		WP_015183292.1	803		Peptidase C14	53-317	YS
					DUF4384	638-731	
		WP_015181297.1	158		Peptidase C14*	8-151	HC
		WP_015211577.1	1571		Peptidase C14*	8-151	HC
					AAA_16	319-477	
					WD40	1328-1353, 1496-1533	
		WP_015180902.1	843		Peptidase C14	3-234	HC
		WP_015186160.1	361		Peptidase C14	111-319	HC
		WP_015211578.1	710		Peptidase C14	12-301	HC
		AFZ17485.1	552	520-542	Peptidase C14	3-201	HC
					GUN4	399-524	
		WP_015184782.1	709		Peptidase C14	3-233	HC
		WP_015180216.1	466		Peptidase C14	4-197	YC
					GUN4	304-435	
41	*Microcystis aeruginosa* FD4	WP_15821602.1	485		Peptidase C14	6-218	HC
					GUN4	341-475	
		WP_158201110.1	691		Peptidase C14	40-277	YS
		WP_158199274.1	616		Peptidase C14*	19-142	HC
		WP_158199615.1	1003		Peptidase C14*	3 to 95	N-
42	*Microcystis aeruginosa* NIES-298	WP_103112467.1	518		Peptidase C14	182-407	HC
		WP_103111476.1	698		Peptidase C14	14-299	HC
		WP_103112730.1	1081		Peptidase C14	3-241	HC
		GBD51629.1	676		Peptidase C14	25-262	YS
		WP_103111461.1	691		Peptidase C14	40-277	YS
		WP_103112637.1	667		Peptidase C14	5-243	HC
					FGE-sulfatase	385-660	
		GBD51846.1	657		Peptidase C14	6-218	HC
					GUN4	511-634	
		WP_103113201.1	616		Peptidase C14*	19-142	H-
43	*Microcystis aeruginosa* NIES-843	WP_012265613.1	547		Peptidase C14*	45-207	HC
		WP_012265065.1	690		Peptidase C14	40-277	YS
		BAG01523.1	83		Peptidase C14*	3 to 77	_
		BAG02469.1	272		Peptidase C14	9-253	HC
44	*Microcystis aeruginosa* NIES-2481	AOC52613	578		Peptidase C14	5-243	HC
		AOC52479.1	675		Peptidase C14	25-257	YS
		WP_066029752.1	690		Peptidase C14	40-272	YS
		WP_046661857.1	1006		Peptidase C14*	3-115	N-
		AOC52706.1	504	458-480	Peptidase C14	36-167	HC
					GUN4	313-444	
45	*Microcystis aeruginosa* PCC 7806SL	ARI83710.1	456		Peptidase C14	46-264	HC
					GUN4	305-445	
		WP_002745414.1	464		Peptidase C14	54-272	HC
					GUN4	313-453	
		WP_002743757.1	698		Peptidase C14	14-299	HC
		WP_036400581.1	671		Peptidase C14	6-218	HC
					GUN4	341-464, 511-629	
		ARI81523.1	675		Peptidase C14	25-257	YS
		WP_036400823.1	690		Peptidase C14	40-272	YS
		WP_002747227.1	935		Peptidase C14	3-235	HC
		WP_002746557.1	622		Peptidase C14	5-243	HC
					FGE-sulfatase	373-615	
46	*Microcystis panniformis* FACHB-1757	AKV65607.1	550	7-26	Peptidase C14	57-276	HC
					GUN4	390-525	

		AKV68101.1	676		Peptidase C14	25-257	YS
		WP_052276698.1	691		Peptidase C14	40-272	YS
		WP_052277029.1	934		Peptidase C14	3-231	HC
		AKV68759.1	929		Peptidase C14	21-226	HC
		AKV66220.1	612		Peptidase C14*	15-138	HC
		WP_052275695.1	616		Peptidase C14*	19-142	HC
		WP_052276893.1	376		Peptidase C14	72-244	HC
47	*Microcystis* sp. MC19	WP_106908538.1	605		Peptidase C14	51-267	HC
					TPR_1	386-417, 557-588	
					TPR_11	358-398	
					TPR_12	487-553, 417-485, 317-383, 282-349	
		WP_106909224.1	690		Peptidase C14	40-277	YS
		WP_106909494.1	623		Peptidase C14	19-236	HC
48	*Microcystis viridis* NIES-102	WP_125732658.1	312		Peptidase C14*	42-204	HC
		BBH37713.1	315		Peptidase C14*	45-207	HC
		WP_125731015.1	526	7-26	Peptidase C14	54-273	HC
					GUN4	375-498	
		BBH42420.1	675		Peptidase C14	25-262	YS
		WP_125732323.1	690		Peptidase C14	40-277	YS
		WP_125730245.1	389		Peptidase C14	9-253	HC
49	*Moorea producens* PAL-8-15-08-1	WP_070395938.1	1518		Peptidase C14	6-260	HC
					WD40	1440-1478	
		WP_070391563.1	770		Peptidase C14	53-321	FS
		WP_070395940.1	1475		Peptidase C14	6-260	HC
					WD40	889-922	
		WP_070392469.1	1238	1199-1218	Peptidase C14	6-279	HC
		WP_070392465.1	1514		Peptidase C14	6-258	HC
					WD40	1305-1343, 963-1001, 1347-1385, 1005-1043, 1131-1169, 921-959, 1047-1085, 1221-1259, 1263-1301, 1089-1127, 1389-1427, 1431-1463, 1180-1217, 885-917	
		WP_070391765.1	1454		Peptidase C14	6-258	HC
					WD40	1337-1375, 972-1009, 1054-1091, 1177-1214, 1095-1132, 1013-1050, 1140-1173, 847-879, 1218-1254, 944-968	
		WP_083305259.1	1752	5-24	Peptidase C14	36-288	HC
					WD40	1125-1163, 1335-1373, 1503-1540, 915-953, 1377-1415, 1419-1457, 1461-1499, 1167-1205, 1212-1247, 957-995, 999-1037, 879-911, 1083-1121, 1293-1331	
		WP_070392841.1	742	684-706	Peptidase C14	403-620	HC
					CHAT	67-338	
		WP_158517387.1	1613	526-548, 753-770	Peptidase C14	6-258	HC
					WD40	1471-1508, 968-1006, 1177-1215, 1219-1257, 926-964, 1093-1131, 1261-1299, 1135-1173, 1345-1383, 1051-1088, 1387-1425, 1429-1467, 848-880, 884-922, 1303-1341, 1010-1047	
		WP_070392891.1	1677		Peptidase C14	5-232	HC
					AAA_16	275-420	
					WD40	973-1011, 1099-1137, 1141-1179, 1267-1305, 889-927, 1057-1095, 1309-1347, 1477-1514, 931-969, 1393-1431, 1015-1053, 1435-1473, 1183-1221, 1351-1389, 1225-1263, 853-885	
		WP_070396394.1	503		Peptidase C14	4-215	QD
		WP_070395648.1	642		Peptidase C14*	16-142	HC
		AOX03865.1	322		Peptidase C14*	7-158	HC
					AAA_16	272-327	
		WP_070393092.1	641		Peptidase C14	6-230	CN
		AOW99620.1	213		Peptidase C14*	7-158	HC
		WP_070394723.1	69		Peptidase C14*	6 to 49	_
		WP_070394975.1	476		Peptidase C14	13-245	HC
50	*Nodularia spumigena* UHCC 0039	WP_107806926.1	713		Peptidase C14	42-290	YS
		WP_107806073.1	1727		Peptidase C14	25-229	HC

					WD40	1481-1519, 1522-1560, 1317-1354, 1153-1191, 1563-1601, 1604-1642, 1358-1395, 1194-1232, 1646-1684, 1235-1273, 1276-1314, 1403-1437, 1121-1149	
		WP_017804406.1	658	381-400	Peptidase C14	5-214	YN
51	*Nostoc azollae* 0708	ADI63760.1	719		Peptidase C14	45-292	YS
		WP_041639652.1	716		Peptidase C14	42-289	YS
		WP_013191329.1	630	358-377	Peptidase C14	5-210	YN
52	*Nostoc flagelliforme* CCNUN1	WP_100897226.1	701		Peptidase C14	42-290	YS
		AUB40246.1	854		Peptidase C14	3-234	HC
					PT	746-771	
		WP_100900790.1	733		Peptidase C14	3-236	HC
		WP_100904073.1	1675		Peptidase C14	5-270	HC
		WP_100898379.1	616	357-376	Peptidase C14	5-228	YN
		AUB42587.1	90		Peptidase C14*	3 to 77	_
		WP_100902396.1	635		Peptidase C14	18-228	HC
		WP_157816981.1	80		Peptidase C14*	2 to 38	_
53	*Nostoc punctiforme* PCC 73102	WP_012412995.1	763		Peptidase C14	45-318	HC
					DUF4384	594-679	
		WP_012412974.1	727		Peptidase C14*	45-273	HC
		WP_012411887.1	650		Peptidase C14	4-198	HC
					GUN4	487-620	
		WP_012409746.1	772	711-729	Peptidase C14	6-283	HC
		WP_012410137.1	841		Peptidase C14	3-231	HC
					Peripla BP_6	492-827	
		WP_012409857.1	924	705-724	Peptidase C14	5-239	HC
					AAA_16	273-396	
					Pentapeptide	771-810, 853-887	
		WP_012411702.1	707		Peptidase C14	42-290	YS
		ACC81713.1	1030		Peptidase C14	3-234	HC
					FGE-sulfatase	844-1065	
		WP_012407604.1	633	357-376	Peptidase C14	5-214	YN
		WP_041562646.1	482		Peptidase C14	13-252	HC
54	*Nostoc* sp. ATCC 53789	RCJ34373.1	912		Peptidase C14	3-235	HC
		WP_114084680.1	670		Peptidase C14	6-278	HC
		WP_114084688.1	622		Peptidase C14	8-258	HC
		RCJ19266.1	644		Peptidase C14	30-280	HC
		WP_114084702.1	844	383-405	Peptidase C14	3-202	HC
					Peripla BP_6	496-830	
		WP_114080469.1	709		Peptidase C14	42-290	YS
		RCJ17787.1	1077		Peptidase C14	3-234	HC
					FGE-sulfatase	842-1070	
		WP_114085540.1	626	357-376	Peptidase C14	5-228	YN
		WP_114081273.1	339		Peptidase C14	8-241	HC
55	*Nostoc* sp. C052	WP_179064679.1	1156		Peptidase C14	3-231	HC
					WD40	1023-1061, 830-868, 796-827, 874-912, 1072-1107	
		WP_179068114.1	827	373-395	Peptidase C14	3-229	HC
					Peripla BP_6	476-813	
		WP_179065887.1	841	379-401	Peptidase C14	3-231	HC
					Peripla BP_6	493-827	
		WP_179066850.1	1559		Peptidase C14	5-228	HC
					Pentapeptide	898-930	
					WD40	969-1007, 1101-1133, 1393-1427, 1227-1259, 1269-1301, 1311-1343, 1479-1511, 1183-1217, 1059-1091, 1017-1049, 1143-1469, 1353-1385	
		WP_179068579.1	706		Peptidase C14	42-288	YS
		WP_179069641.1	1557		Peptidase C14	5-230	HC
					Pentapeptide	895-933	
					WD40	972-1010, 1062-1094, 1314-1346, 1440-1472, 1272-1304, 1398-1430, 1104-1136, 1356-1388, 1146-1178, 1020-1052, 1188-1220, 1482-1514, 1228-1262	
		WP_179068426.1	618	349-368	Peptidase C14	5-212	YN
		WP_179067737.1	1731		Peptidase C14	25-224	HC
					AAA_16	537-689	
					WD40	1564-1601, 1608-1644, 1647-1684, 1139-1176, 1486-1518, 1265-1302, 1524-1559, 1223-1261, 1181-1218, 1306-1344, 1348-1385, 1104-1135, 1442-1475, 1393-1426	
		WP_179064348.1	347		Peptidase C14	9-226	HC

56	*Nostoc* sp. C057	WP_179073233.1	600		Peptidase C14	5-238	HC
					FGE-sulfatase	369-595	
		WP_179077066.1	647		Peptidase C14	3-233	HC
		WP_179074798.1	558		Peptidase C14	3-235	HC
					Clp_N	420-472, 496-544	
		WP_179073995.1	841		Peptidase C14	3-231	HC
					Peripla BP_6	492-827	
		WP_179071513.1	519		Peptidase C14	3-227	HC
					Ribosomal_L12	448-512, 363-427	
		WP_179075309.1	568	406-428	Peptidase C14	3-231	HC
		WP_179077044.1	1509		Peptidase C14	5-200	HC
					AAA_16	279-399	
					MAP7	697-763	
					WD40	1267-1304, 898-935, 1349-1386, 1185-1222, 939-976, 1390-1427, 1103-1140, 1226-1263, 1308-1345, 1144-1181, 980-1017, 1021-1058, 1062-1099, 863-894, 1431-1466	
		WP_179073289.1	709		Peptidase C14	42-290	YS
		WP_179071533.1	629	357-376	Peptidase C14	5-228	YN
		WP_179075617.1	274		Peptidase C14	5-253	HC
57	*Nostoc* sp. CENA543	WP_103136715.1	756		Peptidase C14*	45-218	HC
					DUF4384	593-678	
		WP_103136910.1	694		Peptidase C14	5-238	HC
					FGE-sulfatase	453-689	
		WP_103135695.1	1395		Peptidase C14	3-227	HC
					AAA_16	273-412	
					WD40	1063-1097, 1233-1265, 805-836, 1023-1055, 846-879, 1317-1348, 757-795, 1102-1139, 1275-1307, 973-1005, 1191-1223, 889-920, 1149-1181, 930-963	
		WP_103138996.1	719		Peptidase C14	42-299	YS
		WP_157943191.1	726		Peptidase C14	25-226	HC
					GUN4	547-693	
		AUS99746.1	741		Peptidase C14	40-241	HC
					GUN4	562-708	
		WP_103139965.1	1709		Peptidase C14	25-229	HC
					AAA_16	552-674	
					WD40	1135-1173, 1176-1214, 1504-1542, 1586-1624, 1628-1666, 1340-1378, 1258-1296, 1545-1583, 1217-1255, 1299-1337, 1463-1501, 1103-1131, 1385-1418	
		WP_103140232.1	660	381-400	Peptidase C14	5-209	YN
		WP_103137089.1	1750		Peptidase C14*	81-219	HC
					AAA_16	550-691	
					WD40	1305-1343, 1553-1590, 1637-1675, 1387-1424, 1512-1548, 1595-1632, 1134-1170, 1346-1383, 1223-1259, 1263-1300, 1470-1507, 1676-1717, 1435-1461	
58	*Nostoc* sp. HK-01	BBD63377.1	763		Peptidase C14	45-324	HC
					DUF4384	594-679	
		BBD63356.1	725		Peptidase C14*	43-271	HC
		BBD63193.1	756		Peptidase C14	45-277	HC
					DUF4384	588-673	
		BBD63363.1	738		Peptidase C14	45-307	HC
					DUF4384	583-668	
		BBD57775.1	452		Peptidase C14	51-264	HC
					DUF4384	302-444	
		BBD59748.1	265		Peptidase C14	2-227	QC
		BBD60427.1	713		Peptidase C14	42-291	YS
		BBD60632.1	1238		Peptidase C14	14-214	HC
		BBD62847.1	624		Peptidase C14	5-240	HC
					FGE-sulfatase	375-618	
		BBD60553.1	726		Peptidase C14	25-227	HC
					GUN4	544-688	
		BBD61237.1	646	392-411	Peptidase C14	5-206	YN
		BBD60843.1	325		Peptidase C14	8-258	HC
59	*Nostoc* sp. NIES-3756	WP_067764825.1	674		Peptidase C14	3-232	HC
					FGE-sulfatase	414-666	
		WP_067770252.1	1626		Peptidase C14*	8-147	HC
					WD40	1257-1296, 1035-1064, 1208-1244	
		WP_067768813.1	646		Peptidase C14	3-323	HC
					TPR_12	468-541, 510-624	

		WP_067770240.1	539		Peptidase C14	31-229	HC
		WP_067775128.1	705		Peptidase C14	42-286	YS
		WP_067770228.1	725		Peptidase C14	12-302	HC
		WP_067770250.1	1422		Peptidase C14	14-267	HC
					AAA_16	317-484	
		WP_067768577.1	1611		Peptidase C14	5-202	HC
					WD40	887-925, 929-967, 1310-1346, 1353-1391, 1435-1473, 1224-1262, 1136-1171, 1394-1431, 847-883, 1038-1063, 1082-1116	
		WP_067771937.1	645	369-388	Peptidase C14	5-206	YN
		WP_067771302.1	1705		Peptidase C14*	25-224	HC
					AAA_16	550-696	
					WD40	1582-1620, 1213-1251, 1131-1169, 1624-1162, 1172-1209, 1541-1579, 1459-1496, 1336-1373, 1500-1537, 1254-1292, 1295-1333, 1100-1127, 1381-1415	
		WP_067765039.1	1478		Peptidase C14*	9-158	HC
					AAA_16	270-434	
					WD40	1032-1070, 1074-1112, 909-945, 949-987, 1117-1154, 1200-1238, 991-1028, 1242-1321, 1326-1363, 1156-1196	
60	*Nostoc* sp. PCC 7107	AFY45878.1	218		Peptidase C14	3-197	HC
		AFY45161.1	392	367-389	Peptidase C14	5-231	HC
		AFY41969.1	509		Peptidase C14	51-265	HC
					DUF2808	359-500	
		WP_015115363.1	699		Peptidase C14	3-230	HC
					FGE-sulfatase	447-692	
		AFY44720.1	557		Peptidase C14	3-235	HC
					Clp_N	420-472, 495-542	
		WP_015114428.1	718		Peptidase C14	42-291	YS
		WP_015112508.1	1164		Peptidase C14	5-272	HC
		WP_015113050.1	783		Peptidase C14	56-287	HC
					DUF 2610	701-778	
					Sel1	344-378, 501-536	
		WP_015114275.1	726		Peptidase C14	25-226	HC
					GUN4	544-688	
		WP_015113742.1	646	392-411	Peptidase C14	5-206	YN
61	*Nostoc* sp. PCC 7120 = FACHB-418	WP_010996282.1	1683		Peptidase C14	25-217	HC
					AAA_16	528-665	
					WD40	1229-1266, 1478-1516, 1103-1140, 1271-1309, 1562-1599, 1187-1225, 1436-1474, 1135-1183, 1520-1557, 1312-1349, 1068-1100, 1603-1640, 1395-1431, 1355-1391	
		WP_010997377.1	610		Peptidase C14	5-241	HC
					FGE-sulfatase	368-605	
		WP_010994461.1	1711		Peptidase C14	25-229	HC
					AAA_16	551-697	
					WD40	1630-1668, 1137-1174, 1588-1626, 1342-1379, 1219-1257, 1178-1216, 1465-1503, 1506-1544, 1547-1585, 1301-1339, 1260-1298, 1105-1133, 1424-1462, 1387-1421	
		WP_010994460.1	1747		Peptidase C14	24-219	HC
					AAA_16	550-722	
					WD40	1550-1587, 1633-1671, 1304-1342, 1509-1545, 1128-1165, 1222-1258, 1262-1299, 1591-1628, 1676-1713, 1345-1382, 1467-1504, 1168-1205	
		WP_010999263.1	721		Peptidase C14	42-296	YS
		WP_010999143.1	236		Peptidase C14	3-231	HC
		WP_010997820.1	649	374-393	Peptidase C14	5-206	YN
		WP_096637213.1	650		Peptidase C14	3-231	HC
					FGE-sulfatase	408-629	
62	*Nostoc* sp. PCC 7524	WP_015137385.1	747		Peptidase C14	3-234	HC
		WP_015138745.1	926		Peptidase C14	3-230	HC
					HATPase_c	811-923	
					HisKA	700-765	

		WP_015140169.1	655		Peptidase C14	3-235	HC
		WP_015140776.1	756		Peptidase C14	3-221	HC
					EAL	489-734	
		WP_015137058.1	712		Peptidase C14	38-293	YS
		WP_015138119.1	668		Peptidase C14	3-234	HC
					FGE-sulfatase	424-661	
		WP_015137930.1	668	391-410	Peptidase C14*	5-183	YN
		WP_015138611.1	1711		Peptidase C14	25-229	HC
					AAA_16	552-674	
					WD40	1178-1215, 1137-1175, 1301-1339, 1506-1544, 1630-1668, 1260-1298, 1588-1626, 1342-1380, 1219-1256, 1465-1503, 1547-1585, 1105-1133, 1387-1420	
63	*Nostoc* sp. TCL26-01	WP_179050571.1	597		Peptidase C14	5-238	HC
					FGE-sulfatase	368-593	
		WP_179050384.1	938		Peptidase C14	3-228	HC
					AAA_16	274-413	
					HATPase_c	814-932	
					HisKA	703-768	
		WP_179051001.1	1797		Peptidase C14	4-223	HC
		WP_179047370.1	646		Peptidase C14	3-233	HC
					TPR_12	468-539	
		WP_179049636.1	705		Peptidase C14	42-288	YS
		WP_179048829.1	861		Peptidase C14	3-222	HC
					TPR_11	709-750, 607-648, 746-784, 542-580, 505-546, 811-852, 777-818, 644-682, 474-512, 576-614, 678-716	
		WP_179049723.1	710		Peptidase C14	3-232	HC
					FGE-sulfatase	457-706	
		WP_179049843.1	1779		Peptidase C14	24-219	HC
					AAA_16	550-685	
					WD40	1665-1703, 1582-1619, 1541-1577, 1161-1198, 1335-1373, 1417-1454, 1253-1289, 1220-1383, 1623-1660, 1708-1745, 1499-1536, 1376-1413, 1463-1490	
		WP_179046391.1	1523		Peptidase C14	5-237	HC
					AAA_16	281-450	
					WD40	861-899, 1025-1063, 1353-1391, 984-1022, 943-981, 1394-1431, 1312-1350, 1271-1309, 902-940, 1148-1189, 1107-1145, 825-858, 1230-1268, 1186-1227, 1066-1104	
		WP_179051528.1	1738		Peptidase C14	25-229	HC
					WD40	1164-1201, 1369-1407, 1492-1529, 1246-1284, 1574-1612, 1615-1653, 1533-1571, 1328-1366, 1205-1242, 1657-1695, 1132-1160, 1287-1325, 1414-1447	
		WP_179049831.1	645	378-397	Peptidase C14	5-206	YN
64	*Nostoc* sp. TCL240-02	WP_174708525.1	675		Peptidase C14	5-231	HC
		WP_174711779.1	843		Peptidase C14	3-231	HC
					Peripla BP_6	495-829	
		WP_174708452.1	709		Peptidase C14	42-290	YS
		WP_174711032.1	394		Peptidase C14	146-336	HC
		WP_174710731.1	1499		Peptidase C14	4-222	HC
		WP_174710198.1	631	357-376	Peptidase C14	5-214	YN
65	*Nostoc sphaeroides* CCNUC1	WP_152592918.1	669		Peptidase C14	6-274	HC
		WP_152589559.1	599		Peptidase C14	5-231	HC
					FGE-sulfatase	368-594	
		WP_152590938.1	998	802-821	Peptidase C14*	8-143	HC
					AAA_21	328-565	
					WD40	931-969, 896-927	
		WP_152592919.1	1502		Peptidase C14	8-258	HC
					WD40	1305-1343, 883-921, 925-963, 1179-1217, 1221-1259, 1263-1301, 1347-1384, 967-1005, 1009-1047, 1389-1426, 1143-1175, 844-877, 1092-1132	
		WP_152590348.1	1412		Peptidase C14	3-228	HC

					WD40	1083-1115, 1335-1366, 908-940, 1041-1073, 1209-1241, 782-814, 1167-1199, 1293-1325, 1125-1157, 866-898, 824-856, 1251-1283, 999-1031, 950-989	
		WP_152590945.1	1471		Peptidase C14	12-277	HC
					WD40	1051-1089, 930-968, 1226-1259, 1262-1302, 971-1008, 1092-1130, 1305-1343, 842-879, 1346-1384, 1012-1048	
		WP_152590942.1	1273		Peptidase C14	124-402	HC
		WP_152587949.1	1148		Peptidase C14	3-242	HC
					AAA_16	288-414	
					WD40	884-922, 1060-1098, 852-880, 801-837, 971-1010, 935-968	
		WP_152589074.1	597		Peptidase C14	3-235	HC
					TPR_1	480-511	
					TPR_2	546-576	
					TPR_11	519-559	
		WP_152590036.1	1556		Peptidase C14	5-230	HC
					Pentapeptide	900-932	
					WD40	971-1009, 1271-1303, 1187-1219, 1061-1093, 1145-1177, 1019-1051, 1355-1387, 1103-1135, 1481-1513, 1229-1261, 1439-1471, 1395-1429, 1311-1345	
		QFS45445.1	594		Peptidase C14	1-232	HC
					TPR_1	477-508	
					TPR_2	543-573	
					TPR_11	516-556	
		WP_152589664.1	925	705-724	Peptidase C14	5-239	HC
					AAA_16	273-396	
					Pentapeptide	771-810, 806-844, 831-889	
		QFS42902.1	690		Peptidase C14	18-266	YS
		WP_152588033.1	714		Peptidase C14	18-266	YS
		WP_152591041.1	309		Peptidase C14	8-236	HC
		WP_152590944.1	1536		Peptidase C14	10-276	HC
					AAA_16	326-364	
					WD40	942-980, 1362-1400, 987-1021, 1404-1442, 1320-1358, 1028-1062, 1201-1233, 901-930, 1153-1191, 1119-1149, 1446-1485	
		WP_152591681.1	183		Peptidase C14*	7-167	HC
		WP_152589269.1	618	352-371	Peptidase C14	5-228	YN
		WP_152588402.1	1115		Peptidase C14*	7-167	HC
					WD40	911-949, 953-991, 995-1033, 1037-1074, 875-907	
		WP_152591888.1	527		Peptidase C14	24-240	HC
66	*Nostocales cyanobacterium* HT-58-2	WP_087541861.1	1237	1198-1217	Peptidase C14	6-275	HC
		WP_087545120.1	934		Peptidase C14	3-230	HC
					HATPase_c	812-930	
					HisKA	698-766	
		WP_087541858.1	1475		Peptidase C14*	8-141	HC
					AAA_16	307-461	
					WD40	1141-1173, 969-1006	
		WP_087541860.1	1527		Peptidase C14*	8-141	HC
					WD40	1370-1407, 1157-1194, 1411-1447, 1329-1367, 1451-1488, 1073-1106, 1110-1147, 1238-1276	
		WP_157679552.1	544		Peptidase C14	3-235	HC
					GUN4	397-523	
		WP_087541528.1	833		Peptidase C14	3-231	HC
					Peripla BP_6	485-817	
		WP_087538897.1	976		Peptidase C14	3-234	HC
					PBP-like_2	651-914	
		WP_087538831.1	732		Peptidase C14	42-300	YS
					DUF4384	566-660	
		WP_087539618.1	656	406-428	Peptidase C14*	8-153	HY
					TPR_1	539-570	
					TPR_11	510-551	
					TPR_12	571-637, 479-535	
		WP_087538971.1	821		Peptidase C14	5-242	HC
					FGE-sulfatase	585-814	
					PT	501-530	
		WP_087541567.1	586	274-296	Peptidase C14	5-242	YN

		WP_087537805.1	314		Peptidase C14	8-243	HC
67	*Oscillatoria acuminata* PCC 6304	WP_015149096.1	735		Peptidase C14*	42-199	HC
					DUF4384	580-664	
		WP_015148013.1	755		Peptidase C14	44-300	YS
		WP_015148808.1	1643		Peptidase C14	4-230	HC
					AAA_16	267-410	
					TPR_2	1462-1494	
					WD40	1127-1165, 909-947, 827-865, 868-906, 1038-1076, 997-1035, 791-824, 1338-1369, 1168-1206, 1209-1247, 1080-1124	
		WP_015150419.1	502		Peptidase C14	8-259	HC
		WP_015150990.1	774		Peptidase C14*	3 to 98	H-
					WD40	692-730, 608-646, 567-603, 734-771, 650-688, 480-513	
68	*Oscillatoria nigro-viridis* PCC 7112	WP_015179051.1	279		Peptidase C14	4-254	HC
		WP_015174546.1	1320	1268-1290	Peptidase C14	6-292	HC
		WP_015211748.1	592		Peptidase C14	5-232	HC
					SAVED	394-570	
		WP_015177194.1	723		Peptidase C14	44-298	CS
		WP_015174545.1	1497		Peptidase C14	8-258	HC
					AAA_16	327-491	
					WD40	1054-1091, 1253-1290, 932-969, 1212-1249, 1419-1454, 973-1010, 1294-1331, 897-928, 1375-1412, 1335-1371, 1014-1050, 1095-1131	
69	*Oxynema* sp. AP17	WP_168568882.1	765		Peptidase C14	44-299	YS
		WP_168571075.1	1771		Peptidase C14	2-209	HC
					AAA_16	278-420	
					TPR_16	1549-1616	
					WD40	1201-1232, 1327-1359, 1075-1106, 907-938, 1117-1148, 823-854, 1159-1190, 991-1022, 1243-1274, 1285-1316, 865-896, 1033-1064, 949-980	
		WP_168571596.1	778	389-411	Peptidase C14	3-243	HC
					WD40	656-694, 614-652, 530-568, 740-777, 698-736, 462-526	
		WP_168571220.1	740		Peptidase C14	7-209	YN
70	*Planktothrix agardhii* NIES-204	BBD55940.1	397		Peptidase C14	45-308	HC
		BBD52759.1	1514		Peptidase C14	8-211	HC
					AAA_16	308-459	
					WD40	1062-1100, 1104-1141, 891-925, 1442-1475, 1391-1434, 1158-1190, 945-974, 844-882, 1346-1384, 990-1018, 1021-1028	
		BBD55944.1	231		Peptidase C14*	45-195	HC
		BBD53756.1	425		Peptidase C14	8-246	HC
					TIR_2	284-346	
		BBD54150.1	598		Peptidase C14	5-200	HN
		BBD53578.1	761		Peptidase C14	40-309	YS
		BBD53828.1	707		Peptidase C14	9-299	HC
		BBD52760.1	699		Peptidase C14	12-302	HC
71	*Pleurocapsa* sp. PCC 7327	WP_015143628.1	758		Peptidase C14	44-304	YS
		WP_015142744.1	581	326-348	Peptidase C14*	6-168	YR
72	*Pseudanabaena* sp. ABRG5-3	BBC24079.1	523		Peptidase C14	6-195	HC
					GUN4	361-467	
		WP_126390677.1	724		Peptidase C14*	44-131	H-
					DUF4384	584-666	
		WP_126390659.1	399		Peptidase C14	6-254	HC
		WP_126390655.1	1261		Peptidase C14	941-1188	HC
		WP_126384414.1	564		Peptidase C14	4-229	HC
					FGE-sulfatase	341-558	
		WP_126384803.1	743		Peptidase C14	45-264	HG
					DUF4384	588-669	
		BBC26624.1	323		Peptidase C14	8-253	HC
73	*Pseudanabaena* sp. PCC 7367	WP_015164269.1	780		Peptidase C14*	51-151	H-
					DUF4384	622-705	
74	*Rippkaea orientalis* PCC 8801	WP_012593925.1	711		Peptidase C14	45-254	YS
		WP_012593008.1	795		Peptidase C14	25-229	HC
		WP_012595661.1	445		Peptidase C14	3-208	YC
75	*Rippkaea orientalis* PCC 8802	WP_015783189.1	711		Peptidase C14	45-254	YS
		WP_012796557.1	795		Peptidase C14	25-229	HC
		WP_015784154.1	445		Peptidase C14	3-208	YC
76	*Rivularia* sp. PCC 7116	AFY56798.1	753		Peptidase C14	3-234	HC
					EAL	486-731	
		WP_015117138.1	736		Peptidase C14*	42-179	Y-
		WP_015120048.1	1633		Peptidase C14	25-212	HC

					WD40	1554-1592, 1084-1122, 1417-1508, 1207-1245, 1512-1550, 1430-1468, 1389-1426, 1267-1305, 1166-1204, 1310-1345	
		WP_015116806.1	606	348-370	Peptidase C14	5-240	YN
		WP_015120480.1	723		Peptidase C14	25-239	HC
		WP_015119030.1	1123		Peptidase C14	811-1056	HC
					HEAT_2	673-759	
		WP_015120781.1	423		Peptidase C14	3-196	HC
		WP_015118852.1	98		Peptidase C14*	6 to 97	H-
77	*Stanieria cyanosphaera* PCC 7437	WP_015193329.1	761		Peptidase C14	50-312	YS
		WP_015195638.1	1738		Peptidase C14	20-216	HC
					AAA_16	540-700	
					WD40	1364-1402, 1622-1660, 1201-1239, 1530-1568, 1323-1361, 1160-1198, 1663-1703, 1488-1527, 1081-1113, 1242-1280, 1283-1320, 1120-1157	
		WP_015212076.1	1737		Peptidase C14	14-231	HC
					AAA_16	515-657	
					WD40	1468-1506, 1116-1154, 1157-1195, 1623-1661, 1427-1465, 1664-1702, 1081-1113, 1198-1241, 1509-1546, 1341-1381, 1244-1278	
		WP_015195165.1	1756		Peptidase C14	26-226	HC
					AAA_16	548-670	
					WD40	1515-1552, 1215-1251, 1600-1637, 1560-1597, 1473-1510, 1106-1143, 1647-1681, 1146-1207, 1380-1427, 1685-1723	
		WP_015195646.1	1808		Peptidase C14	14-231	HC
					AAA_16	511-629	
					PD40	1665-1685	
					WD40	1523-1561, 1689-1727, 1170-1208, 1376-1414, 1129-1167, 1730-1768, 1293-1331, 1423-1455, 1564-1603, 1606-1644, 1211-1249, 1088-1126	
78	*Stanieria* sp. NIES-3757	WP_096384021.1	762		Peptidase C14*	49-137	H-
		WP_096384728.1	1756		Peptidase C14	26-226	HC
					AAA_16	548-670	
					WD40	1215-1251, 1600-1638, 1515-1552, 1560-1597, 1473-1510, 1106-1143, 1647-1681, 1146-1207, 1380-1427, 1685-1723	
79	*Synechococcus elongatus* PCC 6301	WP_155813906.1	671		Peptidase C14*	36-117	H-
		BAD79406.1	679		Peptidase C14*	44-125	H-
80	*Synechococcus elongatus* PCC 7942 = FACHB-805	ABB56328.1	671		Peptidase C14*	36-117	H-
		WP_039755479.1	679		Peptidase C14*	44-125	H-
81	*Synechococcus elongatus* UTEX 3055	WP_126147124.1	679		Peptidase C14*	4-125	H-
82	*Synechococcus lividus* PCC 6715	WP_099797743.1	622		Peptidase C14*	37-117	Y-
83	*Synechococcus* sp. JA-2-3B'a(2-13)	WP_011432265.1	732		Peptidase C14	41-270	YG
84	*Synechococcus* sp. NIES-970	WP_096415465.1	659		Peptidase C14*	44-126	Y-
85	*Synechococcus* sp. PCC 6312	WP_015123192.1	671		Peptidase C14*	42-162	Y-
86	*Synechococcus* sp. PCC 7002	WP_012305740.1	668		Peptidase C14*	44-126	Y-
87	*Synechococcus* sp. PCC 7003	WP_065712853.1	667		Peptidase C14*	44-126	Y-
88	*Synechococcus* sp. PCC 7117	WP_065709860.1	668		Peptidase C14*	44-126	Y-
89	*Synechococcus* sp. PCC 7502	WP_015167715.1	707		Peptidase C14	48-283	HG
					DUF4384	556-639	
90	*Synechococcus* sp. PCC 8807	WP_065715717.1	668		Peptidase C14*	44-126	Y-
91	*Synechococcus* sp. PCC 73109	WP_062431693.1	668		Peptidase C14*	44-126	Y-
92	*Synechococcus* sp. JA-3-3Ab	WP_011429560.1	710		Peptidase C14*	41-122	Y-
93	*Synechocystis* sp. PCC 6803	WP_010873177.1	735		Peptidase C14	40-298	YG
94	*Synechocystis* sp. PCC 6714	WP_028947383.1	747		Peptidase C14	60-301	YG
95	*Thermoleptolyngbya* sp. PKUAC-SCTA183	WP_172356754.1	1812		Peptidase C14	5-195	HC
					AAA_16	273-411	

					WD40	1661-1699, 836-867, 1570-1616, 1067-1095, 1628-1655	
		WP_172356502.1	760		Peptidase C14	44-308	LG
		WP_172358241.1	654		Peptidase C14	5-216	YN
		WP_172355153.1	1227		Peptidase C14	3-222	RG
96	*Thermosynechococcus elongatus* BP-1	WP_011057163.1	625		Peptidase C14*	37-116	Y-
97	*Thermosynechococcus elongatus* PKUAC-SCTE542	QLL29622.1	625		Peptidase C14*	37-117	Y-
98	*Thermosynechococcus* sp. CL-1	WP_149820971.1	625		Peptidase C14*	37-117	Y-
99	*Thermosynechococcus vulcanus* NIES-2134	WP_126986228.1	625		Peptidase C14*	37-116	Y-
100	*Tolypothrix* sp. PCC 7910	WP_167726802.1	761		Peptidase C14	53-285	HC
					DUF4384	594-678	
		WP_167726065.1	725		Peptidase C14*	42-218	HC
		WP_167720235.1	620	13-32	Peptidase C14	55-275	HC
					WD40	494-532, 412-450	
		WP_167718607.1	1520		Peptidase C14	8-259	HC
					AAA_16	308-344	
					WD40	1308-1346, 1267-1305, 1151-1189, 1027-1064, 1349-1387, 1226-1264, 1117-1148, 1398-1434, 897-933	
		WP_167718609.1	671		Peptidase C14	6-279	HC
		WP_167718615.1	307		Peptidase C14*	8-141	HC
		WP_167722542.1	949		Peptidase C14	19-244	HC
					AAA_16	290-420	
					HATPase_c	826-947	
					HisKA	715-780	
		WP_167725958.1	516		Peptidase C14	5-238	HN
					GUN4	354-478	
		WP_167725748.1	706		Peptidase C14	42-291	YS
		WP_167719183.1	1667		Peptidase C14	5-237	HC
					AAA_16	281-407	
					WD40	1174-1212, 1338-1376, 1215-1253, 1461-1499, 969-1007, 1051-1089, 1092-1130, 1297-1335, 1502-1540, 1379-1417, 1543-1580, 1010-1048, 1420-1458, 933-966, 1133-1171, 1258-1294	
		WP_167727401.1	662	384-403	Peptidase C14	5-207	YN
		WP_167723876.1	736		Peptidase C14	25-227	HC
					GUN4	549-695	
		WP_167726474.1	624		Peptidase C14	5-240	HC
					FGE-sulfatase	371-616	
		WP_167720638.1	995		Peptidase C14	4-226	HC
					DEAD	282-444	
					Helicase_C	581-658	
101	*Trichodesmium erythraeum* IMS101	ABG51878.1	781	386-408, 454-476	Peptidase C14	4-236	HC
					FGE-sulfatase	548-774	
					Pro-rich	276-379	
		WP_011611670.1	324		Peptidase C14	8-236	HC
		WP_011612181.1	805		Peptidase C14	44-279	YS
		WP_011611472.1	1343		Peptidase C14	1026-1282	HC
					HEAT_2	511-596, 883-968, 476-534	
		WP_011612045.1	1481		Peptidase C14*	7-112	H-
					AAA_16	295-434	
					WD40	1255-1292, 845-882, 886-923, 968-1005, 1091-1128, 1009-1046, 805-841, 927-964, 1050-1087, 1132-1169, 1296-1333, 1173-1210, 1214-1251, 1337-1374	
		ABG51392.1	664		Peptidase C14*	145-265	Y-
					FGE-sulfatase	408-657	
102	*Trichormus variabilis* ATCC 29413	WP_041456762.1	714		Peptidase C14	42-293	YS
		ABA21999.1	717		Peptidase C14	45-296	YS
		WP_011318970.1	1557		Peptidase C14*	5-186	HC
					Pentapeptide	900-936	
					WD40	1104-1136, 1020-1052, 1062-1094, 1146-1178, 1398-1430, 972-1010, 1356-1388, 1440-1472, 1188-1220, 1314-1346, 1272-1304, 1482-1514, 1230-1262, 943-968	

		WP_011320794.1	729		Peptidase C14	25-233	HC
		WP_011319977.1	309		Peptidase C14	8-236	HC
		WP_011319818.1	1760		Peptidase C14	24-224	HC
					AAA_16	550-735	
					WD40	1646-1684, 1522-1558, 1563-1600, 1235-1271, 1317-1355, 1141-1178, 1604-1641, 1358-1395, 1399-1436, 1689-1726,1275-1312, 1445-1472, 1476-1517	
		WP_011316937.1	1686		Peptidase C14*	25-212	HC
					AAA_16	528-665	
					WD40	1232-1269, 1106-1143, 1274-1312, 1565-1602, 1190-1228, 1481-1519, 1148-1186, 1436-1477, 1523-1560, 1071-1103, 1606-1643, 1358-1394, 1398-1434	
		WP_011318971.1	1240		Peptidase C14	5-239	HC
					AAA_16	280-429	
					WD40	1073-1105, 1031-1063, 947-979, 820-853, 989-1021, 863-895, 1115-1146	
		WP_011319817.1	1711		Peptidase C14	25-229	HC
					AAA_16	551-697	
					WD40	1137-1174, 1630-1668, 1342-1379, 1219-1257, 1547-1585, 1465-1503, 1588-1626, 1178-1216, 1506-1544, 1301-1339, 1260-1298, 1105-1133, 1387-1421, 1424-1462	
		WP_011320322.1	649	374-393	Peptidase C14	5-206	YN

* incomplete sequences
