# Supplementary material for: Insights Into the Phylogenetic Distribution, Diversity, Structural Attributes, and Substrate Specificity of Putative Cyanobacterial Orthocaspases
Source: Front Microbiol. 2021 Jul 2;12:682306. doi: 10.3389/fmicb.2021.682306 (PMC8283722; doi:10.3389/fmicb.2021.682306)
Supplement: Supplementary Table 2 — Detail of templates used for model refinement and energy minimization for three homology models of putative orthocaspases of Nostoc sp. PCC 7120. [file Table_2.docx]

Supplementary Table 2: Detail of templates used for model refinement and energy minimization for three homology models of putative orthocaspases of *Nostoc* sp. PCC 7120.

| **S.no.** | **Template details** | | | | | **Model details** | | **RMSD* (Å)** |
| --- | --- | --- | --- | --- | --- | --- | --- | --- |
|  | **PDB id** | **Chain id** | **Sequence identity (%)** | **Query coverage** | **GMQE** | **Accession no.** | **Catalytic dyad** |  |
| 1. | 3uo8.2 | A | 22.33 | 0.87 | 0.57 | WP_010999143.1 | HC | 0.860 |
| 2. | 4afv.1 | A | 25.64 | 0.92 | 0.62 | WP_010999263.1 | YS | 1.206 |
| 3. | 4af8.1 | A | 19.17 | 0.96 | 0.60 | WP_010997820.1 | YN | 0.963 |

*RMSD (Å) is determined upon superimposition of refined (final) model and respective templates using UCSF Chimera
