# Supplementary material for: Insights Into the Phylogenetic Distribution, Diversity, Structural Attributes, and Substrate Specificity of Putative Cyanobacterial Orthocaspases
Source: Front Microbiol. 2021 Jul 2;12:682306. doi: 10.3389/fmicb.2021.682306 (PMC8283722; doi:10.3389/fmicb.2021.682306)
Supplement: Supplementary Table 3 — Structure quality assessment using Ramachandran plot (PROCHECK) and ModFold8. [file Table_3.docx]

Supplementary Table 3: Structure quality assessment using Ramachandran plot and ModFold8

| **S.no** | **Protein model (accession no.)** | **Ramachandran plot (PROCHECK)** | | | | **ModFOLD8** | | |
| --- | --- | --- | --- | --- | --- | --- | --- | --- |
|  |  | **Most favoured (%)** | **Additional allowed (%)** | **Generously allowed (%)** | **Disallowed (%)** | **Confidence** | **P-value** | **Global model quality score** |
| 1. | HC (WP_010999143.1) | 92.5 | 6.5 | 0.5 | 0.5 | CERT | 2.927E-11 | 0.6226 |
| 2. | YN (WP_010997820.1) | 92.0 | 8.0 | 0.0 | 0.0 | CERT | 1.664E-8 | 0.5947 |
| 3. | YS (WP_010999263.1) | 91.1 | 8.5 | 0.0 | 0.4 | CERT | 2.671E-7 | 0.5824 |
