# Supplementary material for: Insights Into the Phylogenetic Distribution, Diversity, Structural Attributes, and Substrate Specificity of Putative Cyanobacterial Orthocaspases
Source: Front Microbiol. 2021 Jul 2;12:682306. doi: 10.3389/fmicb.2021.682306 (PMC8283722; doi:10.3389/fmicb.2021.682306)
Supplement: Supplementary Table 4 — Detailed description of cyanobacterial strains and the number of diverse putative orthocaspases harbored by them. [file Table_4.pdf]

Supplementary Table 4: Detailed description of cyanobacterial strains and the number of diverse putative orthocaspases harboured by them.

Taxonomical group	Morphology	Cyanobacterial strains	Strain abbreviations	Genome Size (mb)	CDS	Genes	Number of different catalytic dyad containing orthocaspase																			
							HC	YS	YN	YG	YH	YD	YQ	YL	YR	NC	LG	RG	YC	FS	QD	CN	QC	CS	HN	HG
Gloeobacterales	Unicellular	*Gloeobacter kilaueensis* JS1	JS1	4.72479	4337	4511	3	-	-	-	-	-	-	-	-	-	-	-	-	-	-	-	-	-	-	-
		Gloeobacter violaceus PCC 7421	PCC 7421	4.65902	4444	4572	6	-	-	-	-	-	-	-	-	-	-	-	-	-	-	-	-	-	-	-
Synechococcales	Unicellular	*Acaryochloris marina* MBIC11017	MBIC11017	8.3616	7046	7579	7	-	-	1	-	-	-	-	-	-	-	-	-	-	-	-	-	-	-	-
		Prochlorococcus marinus str. AS9601	AS9601	1.66989	1845	1899	-	-	-	-	-	-	-	-	-	-	-	-	-	-	-	-	-	-	-	-
		Prochlorococcus marinus str. MIT 9215	MIT 9215	1.73879	1922	1987	-	-	-	-	-	-	-	-	-	-	-	-	-	-	-	-	-	-	-	-
		Prochlorococcus marinus str. MIT 9301	MIT 9301	1.64188	1785	1839	-	-	-	-	-	-	-	-	-	-	-	-	-	-	-	-	-	-	-	-
		Prochlorococcus marinus str. MIT 9303	MIT 9303	2.68268	2997	3136	-	-	-	-	-	-	-	-	-	-	-	-	-	-	-	-	-	-	-	-
		Prochlorococcus marinus str. MIT 9312	MIT 9312	1.7092	1817	1868	-	-	-	-	-	-	-	-	-	-	-	-	-	-	-	-	-	-	-	-
		Prochlorococcus marinus str. MIT 9313	MIT9313	2.41087	2345	2457	-	-	-	-	-	-	-	-	-	-	-	-	-	-	-	-	-	-	-	-
		Prochlorococcus marinus str. MIT 9515	MIT 9515	1.70418	1792	1856	-	-	-	-	-	-	-	-	-	-	-	-	-	-	-	-	-	-	-	-
		Prochlorococcus marinus str. NATL1A	NATL1A	1.86473	1999	2059	-	-	-	-	-	-	-	-	-	-	-	-	-	-	-	-	-	-	-	-
		Prochlorococcus marinus str. NATL2A	NATL2A	1.8429	1953	2010	-	-	-	-	-	-	-	-	-	-	-	-	-	-	-	-	-	-	-	-
		Prochlorococcus marinus subsp. marinus str. CCMP1375	CCMP1375	1.75108	1843	1897	-	-	-	-	-	-	-	-	-	-	-	-	-	-	-	-	-	-	-	-
		Prochlorococcus marinus subsp. pastoris str. CCMP1986	MED4	1.65799	1855	1908	-	-	-	-	-	-	-	-	-	-	-	-	-	-	-	-	-	-	-	-
		Prochlorococcus sp. MIT 0604	MIT 0604	1.78006	1921	1979	-	-	-	-	-	-	-	-	-	-	-	-	-	-	-	-	-	-	-	-
		Prochlorococcus sp. MIT 0801	MIT 0801	1.9292	2034	2111	-	-	-	-	-	-	-	-	-	-	-	-	-	-	-	-	-	-	-	-
		Synechococcus elongatus PCC 6301 *	PCC 6301	2.69625	2619	2719	-	-	-	-	-	-	-	-	-	-	-	-	-	-	-	-	-	-	-	-
		Synechococcus elongatus PCC 7942 = FACHB-805 *	PCC 7942	2.74227	2685	2756	-	-	-	-	-	-	-	-	-	-	-	-	-	-	-	-	-	-	-	-
		Synechococcus elongatus UTEX 3055 *	UTEX 3055	2.88122	2837	2919	-	-	-	-	-	-	-	-	-	-	-	-	-	-	-	-	-	-	-	-
		Synechococcus lividus PCC 6715 *	PCC 6715	2.65974	2246	2592	-	-	-	-	-	-	-	-	-	-	-	-	-	-	-	-	-	-	-	-
		Synechococcus sp. CB0101	CB0101	2.78965	2931	3074	-	-	-	-	-	-	-	-	-	-	-	-	-	-	-	-	-	-	-	-
		Synechococcus sp. CC9311	CC9311	2.60675	2676	2752	-	-	-	-	-	-	-	-	-	-	-	-	-	-	-	-	-	-	-	-
		Synechococcus sp. CC9605	CC9605	2.51066	2641	2756	-	-	-	-	-	-	-	-	-	-	-	-	-	-	-	-	-	-	-	-
		Synechococcus sp. CC9902	CC9902	2.23483	2338	2405	-	-	-	-	-	-	-	-	-	-	-	-	-	-	-	-	-	-	-	-
		Synechococcus sp. JA-2-3B'a(2-13)	JA-2-3B'a(2-13)	3.04668	2628	2830	-	-	-	1	-	-	-	-	-	-	-	-	-	-	-	-	-	-	-	-
		Synechococcus sp. JA-3-3Ab *	JA-3-3Ab	2.93277	2569	2806	-	-	-	-	-	-	-	-	-	-	-	-	-	-	-	-	-	-	-	-
		Synechococcus sp. KORDI-100	KORDI-100	2.789	2846	2935	-	-	-	-	-	-	-	-	-	-	-	-	-	-	-	-	-	-	-	-
		Synechococcus sp. KORDI-49	KORDI-49	2.58581	2592	2671	-	-	-	-	-	-	-	-	-	-	-	-	-	-	-	-	-	-	-	-
		Synechococcus sp. KORDI-52	KORDI-52	2.57207	2631	2729	-	-	-	-	-	-	-	-	-	-	-	-	-	-	-	-	-	-	-	-
		Synechococcus sp. NIES-970 *	NIES-970	3.12386	2864	2958	-	-	-	-	-	-	-	-	-	-	-	-	-	-	-	-	-	-	-	-
		Synechococcus sp. PCC 6312 *	PCC 6312	3.7205	3529	3695	-	-	-	-	-	-	-	-	-	-	-	-	-	-	-	-	-	-	-	-
		Synechococcus sp. PCC 7002 *	PCC 7002	3.40993	3143	3239	-	-	-	-	-	-	-	-	-	-	-	-	-	-	-	-	-	-	-	-
		Synechococcus sp. PCC 7003 *	PCC 7003	3.34509	3069	3182	-	-	-	-	-	-	-	-	-	-	-	-	-	-	-	-	-	-	-	-
		Synechococcus sp. PCC 7117 *	PCC 7117	3.4321	3157	3274	-	-	-	-	-	-	-	-	-	-	-	-	-	-	-	-	-	-	-	-
		Synechococcus sp. PCC 73109 *	PCC 73109	3.29868	3029	3128	-	-	-	-	-	-	-	-	-	-	-	-	-	-	-	-	-	-	-	-
		Synechococcus sp. PCC 7502	PCC 7502	3.58373	3437	3685	-	-	-	-	-	-	-	-	-	-	-	-	-	-	-	-	-	-	-	1
		Synechococcus sp. PCC 8807	PCC 8807	3.30409	3084	3183	-	-	-	-	-	-	-	-	-	-	-	-	-	-	-	-	-	-	-	-
		Synechococcus sp. RCC307	RCC307	2.22491	2388	2457	-	-	-	-	-	-	-	-	-	-	-	-	-	-	-	-	-	-	-	-
		Synechococcus sp. RSCCF101	RSCCF101	2.98485	2696	2886	-	-	-	-	-	-	-	-	-	-	-	-	-	-	-	-	-	-	-	-
		Synechococcus sp. SynAce01	SynAce01	2.75063	2732	2881	-	-	-	-	-	-	-	-	-	-	-	-	-	-	-	-	-	-	-	-
		Synechococcus sp. WH 7803	WH 7803	2.36698	2456	2528	-	-	-	-	-	-	-	-	-	-	-	-	-	-	-	-	-	-	-	-
		Synechococcus sp. WH 8101	WH 8101	2.63029	2732	2818	-	-	-	-	-	-	-	-	-	-	-	-	-	-	-	-	-	-	-	-
		Synechococcus sp. WH 8102	WH 8102	2.43443	2569	2679	-	-	-	-	-	-	-	-	-	-	-	-	-	-	-	-	-	-	-	-
		Synechococcus sp. WH 8103	WH 8103	2.42969	2552	2650	-	-	-	-	-	-	-	-	-	-	-	-	-	-	-	-	-	-	-	-
		Synechococcus sp. WH 8109	WH 8109	2.11151	2212	2327	-	-	-	-	-	-	-	-	-	-	-	-	-	-	-	-	-	-	-	-
		Synechocystis sp. PCC 6714	PCC 6714	3.73889	3312	3499	-	-	-	1	-	-	-	-	-	-	-	-	-	-	-	-	-	-	-	-
		Synechocystis sp. PCC 6803	PCC 6803	3.94702	3559	3699	-	-	-	1	-	-	-	-	-	-	-	-	-	-	-	-	-	-	-	-
		Thermosynechococcus elongatus BP-1 *	BP-1	2.59386	2423	2531	-	-	-	-	-	-	-	-	-	-	-	-	-	-	-	-	-	-	-	-
		Thermosynechococcus elongatus PKUAC-SCTE542 *	PKUAC-SCTE542	2.64873	1625	2617	-	-	-	-	-	-	-	-	-	-	-	-	-	-	-	-	-	-	-	-
		Thermosynechococcus sp. CL-1 *	CL-1	2.64782	2466	2597	-	-	-	-	-	-	-	-	-	-	-	-	-	-	-	-	-	-	-	-
		Thermosynechococcus vulcanus NIES-2134 *	NIES-2134	2.57127	2396	2502	-	-	-	-	-	-	-	-	-	-	-	-	-	-	-	-	-	-	-	-
		Cyanobium gracile PCC 6307	PCC 6307	3.34236	3248	3403	-	-	-	-	-	-	-	-	-	-	-	-	-	-	-	-	-	-	-	-
		Cyanobium sp. NIES-981	NIES-981	3.02155	2883	3057	-	-	-	-	-	-	-	-	-	-	-	-	-	-	-	-	-	-	-	-
	Filamentous	*Halomicronema hongdechloris* C2206	C2206	5.57784	4661	5115	9	-	1	1	-	-	-	-	-	-	-	-	-	-	-	-	-	-	-	-
		Leptolyngbya boryana NIES-2135	NIES-2135	7.23367	6491	6757	8	-	1	1	-	-	-	-	-	1	-	-	-	-	-	-	-	-	-	-
		Leptolyngbya sp. NIES-3755	NIES-3755	6.76166	6350	6603	5	-	2	-	-	-	-	-	-	-	-	-	-	-	-	-	-	-	-	-
		Leptolyngbya sp. O-77	O-77	5.48026	4383	4729	2	-	1	-	-	-	-	-	-	-	1	1	-	-	-	-	-	-	-	-
		Leptolyngbya sp. PCC 7376	**PCC 7376**	5.12595	4190	4624	8	-	1	-	-	-	-	-	-	-	-	-	-	-	-	-	-	-	-	-
		Pseudanabaena sp. ABRG5-3	ABRG5-3	5.65797	4924	5115	5	-	-	-	-	-	-	-	-	-	-	-	-	-	-	-	-	-	-	1
		Pseudanabaena sp. PCC 7367 *	PCC 7367	4.88568	3823	3952	-	-	-	-	-	-	-	-	-	-	-	-	-	-	-	-	-	-	-	-
		Thermoleptolyngbya sp. PKUAC-SCTA183	PKUAC-SCTA183	5.5251	4404	4590	1	-	1	-	-	-	-	-	-	-	1	1	-	-	-	-	-	-	-	-
Chroococcales	Unicellular	*Chondrocystis* sp. NIES-4102	NIES-4102	5.00252	4198	4367	1	1	-	-	-	-	-	-	-	-	-	-	-	-	-	-	-	-	-	-

		Crocosphaera subtropica ATCC 51142	ATCC 51142	5.46038	4891	5043	2	1	-	-	-	-	2	-	-	-	-	-	-	-	-	-	-	-	-	-
		Cyanobacterium aponinum PCC 10605	PCC 10605	4.17697	3402	3580	2	-	-	-	-	-	-	-	-	-	-	-	-	-	-	-	-	-	-	-
		Cyanobacterium sp. HL-69	HL-69	3.29695	2947	3099	1	-	-	-	-	-	-	-	-	-	-	-	-	-	-	-	-	-	-	-
		Cyanobacterium stanieri PCC 7202	PCC 7202	3.16338	2837	2941	1	-	-	-	-	-	-	-	-	-	-	-	-	-	-	-	-	-	-	-
		Dactylococcopsis salina PCC 8305 *	PCC 8305	3.78101	3444	3701	-	-	-	-	-	-	-	-	-	-	-	-	-	-	-	-	-	-	-	-
		Euhalothece natronophila Z-M001	Z-M001	3.52431	3257	3466	1	1	-	-	-	-	-	-	-	-	-	-	-	-	-	-	-	-	-	-
		Geminocystis sp. NIES-3708 *	NIES-3708	4.04208	3406	3596	-	-	-	-	-	-	-	-	-	-	-	-	-	-	-	-	-	-	-	-
		Geminocystis sp. NIES-3709 *	NIES-3709	4.42606	3591	3833	-	-	-	-	-	-	-	-	-	-	-	-	-	-	-	-	-	-	-	-
		Gloeocapsa sp. PCC 7428	PCC 7428	5.88271	4987	5238	1	1	-	-	-	-	-	-	1	-	-	-	-	-	-	-	-	-	-	-
		Gloeothece citriformis PCC 7424	PCC 7424	6.55417	5544	5850	5	1	-	-	-	-	-	-	-	-	-	-	-	-	-	-	-	-	-	-
		Gloeothece verrucosa PCC 7822	PCC 7822	7.84195	6619	6992	2	1	-	-	-	-	-	-	-	-	-	-	-	-	-	-	-	-	-	-
		Halothece sp. PCC 7418	PCC 7418	4.17917	3713	3902	1	1	-	-	-	-	-	-	-	-	-	-	-	-	-	-	-	-	-	-
		Microcystis aeruginosa FD4	FD4	5.49311	4677	5445	1	1	-	-	-	-	-	-	-	-	-	-	-	-	-	-	-	-	-	-
		Microcystis aeruginosa NIES-2481	NIES-2481	4.44055	3883	4213	2	2	-	-	-	-	-	-	-	-	-	-	-	-	-	-	-	-	-	-
		Microcystis aeruginosa NIES-298	NIES-298	5.01508	4352	4755	5	2	-	-	-	-	-	-	-	-	-	-	-	-	-	-	-	-	-	-
		Microcystis aeruginosa NIES-843	NIES-843	5.84279	4969	5680	1	1	-	-	-	-	-	-	-	-	-	-	-	-	-	-	-	-	-	-
		Microcystis aeruginosa PCC 7806SL	PCC 7806SL	5.13934	4363	4886	6	2	-	-	-	-	-	-	-	-	-	-	-	-	-	-	-	-	-	-
		Microcystis panniformis FACHB-1757	FACHB-1757	5.68684	4577	5570	4	2	-	-	-	-	-	-	-	-	-	-	-	-	-	-	-	-	-	-
		Microcystis sp. MC19	MC19	5.02024	4383	4820	2	1	-	-	-	-	-	-	-	-	-	-	-	-	-	-	-	-	-	-
		Microcystis viridis NIES-102	NIES-102	5.8742	4807	5698	2	2	-	-	-	-	-	-	-	-	-	-	-	-	-	-	-	-	-	-
		Rippkaea orientalis PCC 8801	PCC 8801	4.78769	4299	4517	1	1	-	-	-	-	-	-	-	-	-	-	1	-	-	-	-	-	-	-
		Rippkaea orientalis PCC 8802	PCC 8802	4.80335	4374	4561	1	1	-	-	-	-	-	-	-	-	-	-	1	-	-	-	-	-	-	-
Pleurocapsales	Unicellular	*Pleurocapsa* sp. PCC 7327	PCC 7327	4.98682	4226	4553	-	1	-	-	-	-	-	-	-	-	-	-	-	-	-	-	-	-	-	-
		Stanieria cyanosphaera PCC 7437	PCC 7437	5.54499	4776	4998	4	1	-	-	-	-	-	-	-	-	-	-	-	-	-	-	-	-	-	-
		Stanieria sp. NIES-3757	NIES-3757	5.46331	4668	4926	1	-	-	-	-	-	-	-	-	-	-	-	-	-	-	-	-	-	-	-
Chroococcidiopsidales	Unicellular	*Chroococcidiopsis thermalis* PCC 7203	PCC 7203	6.6894	5716	5950	4	1	-	-	-	-	-	-	-	-	-	-	-	-	-	-	-	-	-	-
Oscillatoriales	Unicellular	*Cyanothece* sp. PCC 7425	PCC 7425	5.78611	5194	5402	-	-	-	1	-	-	-	-	-	-	-	-	-	-	-	-	-	-	-	-
	Filamentous	*Crinalium epipsammum* PCC 9333	PCC 9333	5.62041	4760	5031	4	-	1	-	-	-	-	-	-	-	-	-	-	-	-	-	-	-	-	-
		Geitlerinema sp. PCC 7407	PCC 7407	4.68111	3760	3893	3	-	-	-	-	-	-	-	-	-	-	-	-	-	-	-	-	-	-	-
		Limnospira fusiformis SAG 85.79	SAG 85.79	6.42369	5419	6130	3	1	1	-	-	-	-	-	-	-	-	-	-	-	-	-	-	-	-	-
		Microcoleus sp. PCC 7113	PCC 7113	7.96651	6364	6719	8	1	-	-	-	-	-	-	-	-	-	-	1	-	-	-	-	-	-	-
		Moorea producens PAL-8-15-08-1	PAL-8-15-08-1	9.70866	7469	8089	10	-	-	-	-	-	-	-	-	-	-	-	-	1	1	1	-	-	-	-
		Oscillatoria acuminata PCC 6304	PCC 6304	7.80427	5783	6061	2	1	-	-	-	-	-	-	-	-	-	-	-	-	-	-	-	-	-	-
		Oscillatoria nigro-viridis PCC 7112	PCC 7112	8.27225	6200	6896	4	-	-	-	-	-	-	-	-	-	-	-	-	-	-	-	-	1	-	-
		Oxynema sp. AP17	AP17	6.35775	4801	5069	2	1	1	-	-	-	-	-	-	-	-	-	-	-	-	-	-	-	-	-
		Planktothrix agardhii NIES-204	NIES-204	5.08413	4492	4547	5	1	-	-	-	-	-	-	-	-	-	-	-	-	-	-	-	-	1	-
		Trichodesmium erythraeum IMS101	IMS101	7.75011	4622	5217	3	1	-	-	-	-	-	-	-	-	-	-	-	-	-	-	-	-	-	-
Nostocales	Heterocytous	*Anabaena cylindrica* PCC 7122	PCC 7122	7.06328	5747	6151	1	1	1	-	-	-	-	-	-	-	-	-	-	-	-	-	-	-	-	-
		Anabaena sp. 90	90	5.30567	4520	4918	5	1	1	-	-	-	-	-	-	-	-	-	-	-	-	-	-	-	-	-
		Anabaena sp. WA102	WA102	5.78203	4927	5370	2	-	1	-	-	-	-	-	-	-	-	-	-	-	-	-	-	-	-	-
		Calothrix sp. 336/3	336/3	6.42013	5266	5485	9	1	1	-	-	-	-	-	-	-	-	-	-	-	-	-	-	-	-	-
		Calothrix sp. NIES-2098	NIES-2098	8.87863	7082	7386	11	1	1	-	-	-	-	-	-	-	-	-	-	-	-	-	-	-	-	-
		Calothrix sp. NIES-3974	NIES-3974	5.98588	4508	4744	1	-	1	-	1	-	-	-	-	-	-	-	-	-	-	-	-	-	-	-
		Calothrix sp. NIES-4071	NIES-4071	12.0493	9858	10691	11	2	1	-	-	-	-	-	-	-	-	-	-	-	-	-	-	-	-	-
		Calothrix sp. NIES-4101	NIES-4101	7.58683	6434	6502	11	1	1	-	-	1	-	-	-	-	-	-	-	-	-	-	-	-	-	-
		Calothrix sp. PCC 6303	PCC 6303	6.96039	5437	5723	1	-	-	-	-	-	-	-	-	-	-	-	-	-	-	-	-	-	-	-
		Calothrix sp. PCC 7507	PCC 7507	7.02322	5778	6078	7	1	1	-	-	-	-	-	-	-	-	-	-	-	-	-	-	-	-	-
		Nodularia spumigena UHCC 0039	UHCC 0039	5.38661	4498	4814	1	1	1	-	-	-	-	-	-	-	-	-	-	-	-	-	-	-	-	-
		Nostoc azollae 0708	708	5.48614	3548	5354	-	2	1	-	-	-	-	-	-	-	-	-	-	-	-	-	-	-	-	-
		Nostoc flagelliforme CCNUN1	CCNUN1	10.2312	8291	9654	4	1	1	-	-	-	-	-	-	-	-	-	-	-	-	-	-	-	-	-
		Nostoc punctiforme PCC 73102	PCC 73102	9.05919	6989	7621	7	1	1	-	-	-	-	-	-	-	-	-	-	-	-	-	-	-	-	-
		Nostoc sp. ATCC 53789	ATCC 53789	8.65373	6847	7360	7	1	1	-	-	-	-	-	-	-	-	-	-	-	-	-	-	-	-	-
		Nostoc sp. C052	C052	9.87983	7645	8337	7	1	1	-	-	-	-	-	-	-	-	-	-	-	-	-	-	-	-	-
		Nostoc sp. C057	C057	9.23445	7347	8058	8	1	1	-	-	-	-	-	-	-	-	-	-	-	-	-	-	-	-	-
		Nostoc sp. CENA543	CENA543	7.20764	5818	6053	5	1	1	-	-	-	-	-	-	-	-	-	-	-	-	-	-	-	-	-
		Nostoc sp. HK-01	NIES-2109	7.5277	6424	6534	8	1	1	-	-	-	-	-	-	-	-	-	-	-	-	-	1	-	-	-
		Nostoc sp. NIES-3756	NIES-3756	6.98757	5771	6031	6	1	1	-	-	-	-	-	-	-	-	-	-	-	-	-	-	-	-	-
		Nostoc sp. PCC 7107	PCC 7107	6.32982	5197	5464	8	1	1	-	-	-	-	-	-	-	-	-	-	-	-	-	-	-	-	-
		Nostoc sp. PCC 7120 = FACHB-418	PCC 7120	7.21179	5776	6109	6	1	1	-	-	-	-	-	-	-	-	-	-	-	-	-	-	-	-	-
		Nostoc sp. PCC 7524	PCC 7524	6.71887	5392	5622	6	1	-	-	-	-	-	-	-	-	-	-	-	-	-	-	-	-	-	-
		Nostoc sp. TCL240-02	TCL240-02	7.88725	6200	6765	4	1	1	-	-	-	-	-	-	-	-	-	-	-	-	-	-	-	-	-
		Nostoc sp. TCL26-01	TCL26-01	7.38768	5999	6419	9	1	1	-	-	-	-	-	-	-	-	-	-	-	-	-	-	-	-	-
		Nostoc sphaeroides CCNUC1	CCNUC1	9.51639	7590	8625	14	2	1	-	-	-	-	-	-	-	-	-	-	-	-	-	-	-	-	-
		Nostocales cyanobacterium HT-58-2	HT-58-2	7.89957	6195	6596	7	1	1	-	-	-	-	-	-	-	-	-	-	-	-	-	-	-	-	-
		Rivularia sp. PCC 7116	PCC 7116	8.72877	6441	6724	5	-	1	-	-	-	-	-	-	-	-	-	-	-	-	-	-	-	-	-
		Tolypothrix sp. PCC 7910	PCC 7910	8.76153	6699	7070	9	1	1	-	-	-	-	-	-	-	-	-	-	-	-	-	-	-	1	-

		Trichormus variabilis ATCC 29413	ATCC 29413	7.10575	5717	5943	5	2	1	-	-	-	-	-	-	-	-	-	-	-	-	-	-	-	-	-
		Dolichospermum flos-aquae CCAP 1403/13F	CCAP 1403/13f	5.22068	4457	4947	2	-	1	-	-	-	-	-	-	-	-	-	-	-	-	-	-	-	-	-
		Dolichospermum sp. UHCC 0315A	UHCC 0315A	5.64202	4699	5218	2	1	1	-	-	-	-	-	-	-	-	-	-	-	-	-	-	-	-	-
	Heterocytous-branched	*Fischerella* sp. NIES-3754	NIES-3754	5.82686	4584	4911	2	1	1	-	-	-	-	-	-	-	-	-	-	-	-	-	-	-	-	-

NOTE: * the cyanobacterial strains consisted of only partial or incomplete sequence(s) of Peptidase C14 domain of orthocaspases
